# Metagenomic Analysis of the Buccal Microbiome by Nanopore Sequencing Reveals Structural Differences in the Microbiome of a Patient with Molar Incisor Hypomineralization (MIH) Compared to a Healthy Child—Case Study

**DOI:** 10.3390/ijms252313143

**Published:** 2024-12-06

**Authors:** Wojciech Tynior, Małgorzata Kłósek, Silvia Salatino, Piotr Cuber, Dorota Hudy, Dariusz Nałęcz, Yuen-Ting Chan, Carla Gustave, Joanna Katarzyna Strzelczyk

**Affiliations:** 1 Department of Medical and Molecular Biology, Faculty of Medical Sciences in Zabrze, Medical University of Silesia in Katowice, 19 Jordana St., 41-808 Zabrze, Poland; 2 Department of Microbiology and Immunology, Faculty of Medical Sciences in Zabrze, Medical University of Silesia in Katowice, 19 Jordana St., 41-808 Zabrze, Poland; 3 Molecular Biology Laboratories, Science and Innovation Platforms, Natural History Museum, London SW7 5BD, UK; 4 Department of Otolaryngology and Maxillofacial Surgery, St. Vincent De Paul Hospital, 1 Wójta Radtkego St., 81-348 Gdynia, Poland

**Keywords:** molar-incisor hypomineralization (MIH), buccal microbiome, pathogens, nanopore sequencing, bacteria, buccal swabs

## Abstract

Molar incisor hypomineralization (MIH) is a qualitative developmental defect that affects the enamel tissue of permanent molars and can also occur in permanent incisors. Enamel affected by MIH has reduced hardness, increased porosity, and a higher organic content than unaffected enamel. These characteristics predispose the enamel to accumulation of bacteria and a higher prevalence of caries lesions. Through a groundbreaking metagenomic analysis of the buccal mucosal sample from a patient with MIH, we explored the intricacies of its microbiome compared to a healthy control using state-of-the-art nanopore long-read sequencing. Out of the 210 bacterial taxa identified in the MIH microbiome, we found *Streptococcus* and *Haemophilus* to be the most abundant genera. The bacteria with the highest read counts in the patient with MIH included *Streptococcus mitis*, *Haemophilus parainfluenzae*, *Streptococcus pneumoniae*, *Rothia dentocariosa*, and *Gemella haemolysans*. Our results revealed a striking contrast between healthy and MIH affected children, with a higher dominance and number of pathogenic species (*S. pneumoniae*, *H. influenzae*, and *N. meningitidis*) and reduced diversity in the MIH-affected patient. This distinct microbial profile not only sheds light on MIH-affected patients, but paves the way for future research, inspiring deeper understanding and larger scale studies.

## 1. Introduction

Molar incisor hypomineralization (MIH) is a qualitative developmental defect that affects the enamel tissue of permanent teeth. The prevalence of this condition varies worldwide. The global prevalence is 14.2% and differs between continents and countries [1,2]. According to the European Academy of Paediatric Dentistry (EAPD), MIH is diagnosed when one of the first permanent molars is affected [3]. It can also appear on permanent incisors. The severity of MIH can vary. Clinicians may observe wide range of opacities in affected teeth, from white and creamy to yellow and brown. In severe cases, posteruptive enamel breakdown (PEB) may be detected. Due to the nature of the disease, varied dental treatments may be recommended, e.g., non-invasive treatment, a variety of atypical fillings, and, in the most severe cases, tooth extraction [3,4,5].

The enamel affected by MIH is less hard, more porous, and has a higher organic content than that of unaffected individuals [6]. The mechanical properties of the enamel predispose it to the accumulation of pathogens and the increased prevalence of caries lesions [7,8]. However, the specificity of the oral cavity is considered a diverse environment, and includes the tooth surface, gingival sulcus, and oral epithelium, with complex interspecies and pathogen-host interactions [9,10].

The oral mucosa consists of a lamina propria covered by epithelium, which histologically belongs to the stratified squamous epithelium. The inner buccal surface is covered by non-keratinized squamous epithelium [11]. The microbiota of the buccal mucosa is characterized by stability, making it an ideal site for our study. The microbiota consists of both the physiological flora and pathogenic microorganisms living in and on the human body. It varies depending on the niches it inhabits and the individual’s health status. The microbiome refers to the complete collection of genomes that make up the microbiota. The oral cavity contains various microenvironments for bacteria, archaea, or eukaryotes colonization, including the teeth (supragingival and subgingival plaque), gingival pockets, tongue, palate, or buccal mucosa [9,10]. Each niche is colonized by slightly different microbial species due to differences in environmental factors such as oxidation-reduction potential, nutrient availability, saliva pH, or ligands for bacterial adhesins. The predominant bacteria cultured from the cheek (buccal mucosa) are streptococci, especially mitis group, and other Gram-positive cocci like *Gemella* spp. and *Granulicatella* spp. Other bacteria that can be present in the buccal mucosa are *Veillonella* spp. (Gram-negative cocci) and the Gram-negative rods *Prevotella* spp. and *Haemophilus parainfluenzae*. *Streptococcus oralis* is found in dental plaque and *Streptococcus infantis* on the tongue dorsum. Predominant species in the supragingival plaque are *Streptococcus mutans*, *S. salivarius*, *S. mitis*, and *Lactobacillus* spp. In the subgingival plaque, *Actinobacillus* spp., *Campylobacter* spp., *Fusobacterium nucleatum*, and *Porphyromonas gingivalis* are mostly found. Examination of the microbiome in healthy humans revealed the presence of fungi, like *Candida* spp., *Saccharomyces* spp., *Penicillium* spp., *Aspergillus* spp., and *Malassezia* spp., as well as archaea and bacteriophages [12,13,14]. Changes in the microbiome, known as dysbiosis, can lead to pathological conditions in the oral cavity, such as caries, gingivitis, or periodontitis [2,15].

The aim of this research was to analyse the buccal mucosa microbiome in a patient with MIH and in a matched healthy child using a novel approach—that is, nanopore sequencing and metagenomic analysis—never previously used to study this disease, to the best of our knowledge.

## 2. Results

ONT sequencing of the epithelium control and MIH samples resulted in 31,152,066 reads and 25,079,108 reads, respectively. Following a series of trimming, quality filtering, and host removal steps, sequencing reads underwent taxonomic classification using the k-mer based tool Kraken2. Key sequencing and filtering metrics for each of the above-mentioned bioinformatic analysis workflow steps are shown in Table 1.

**Figure 1:**
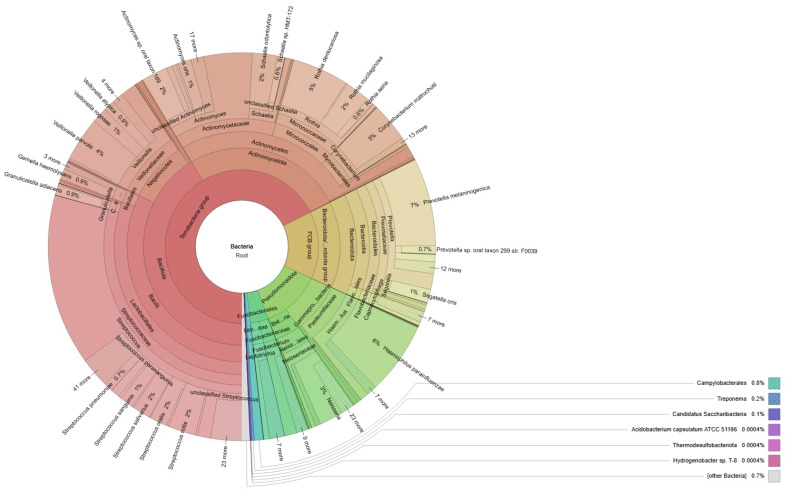
Taxonomic distribution of the bacterial reads in healthy child classified by Kraken2.

**Figure 2:**
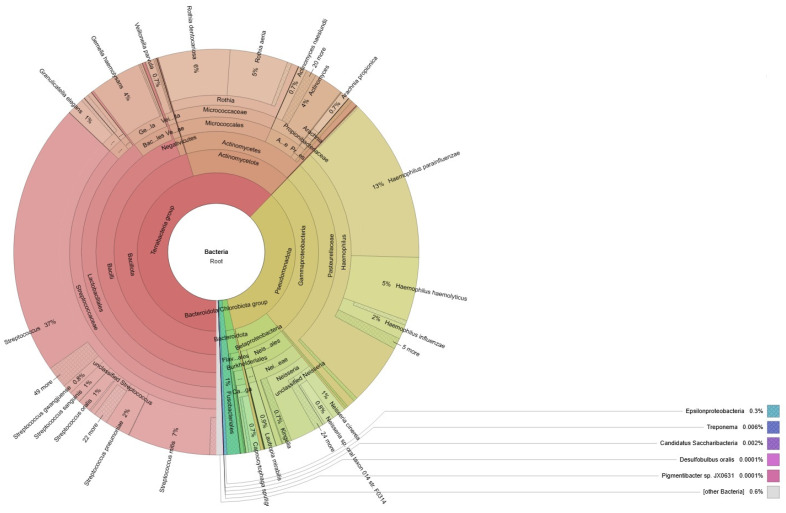
Taxonomic distribution of the bacterial reads in MIH-affected child classified by Kraken2.

A visual representation of the bacteria identified through our bioinformatic analysis is shown in Figure 1 and Figure 2 through Krona plots, a set of interactive radial space-filling charts used to display the abundance and hierarchy of taxonomic assignments from a metagenomics experiment. Interactive Krona plots from both patients are presented in Supplementary Materials (Figures S1 and S2).

**Figure 3:**
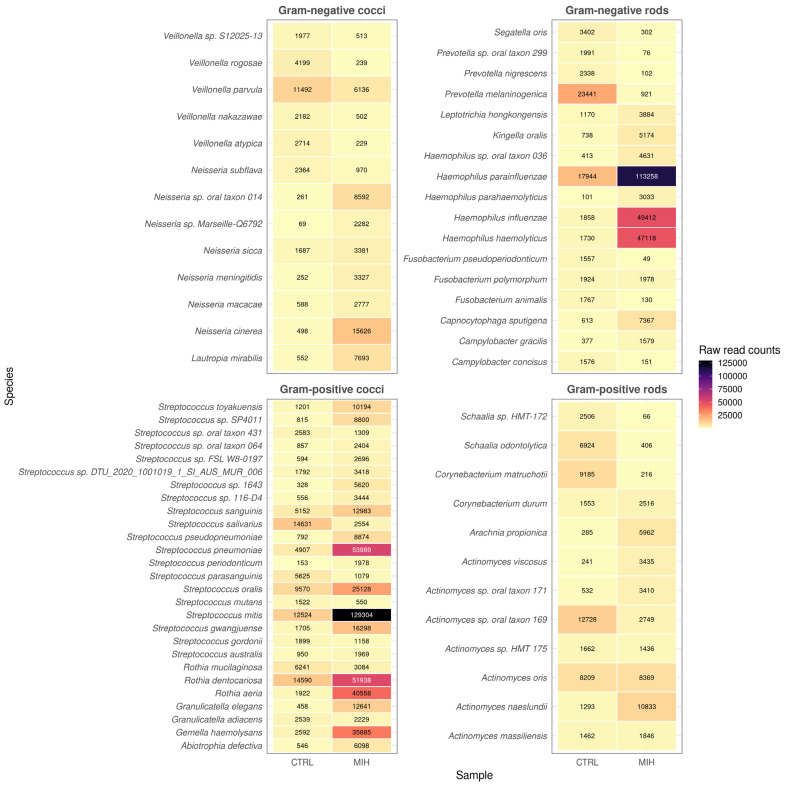
Species-level abundance estimates for the healthy child and MIH patient, grouped by Gram staining and bacterial morphology.

Kraken2 results were refined using Bracken to generate accurate species-level abundance estimates within the two microbial samples. In total, 210 species of microbes were identified in a single patient with MIH, and 223 in a healthy individual. All were bacteria from different groups. We decided to apply a cut-off of 1500 minimum reads per species (three times higher than the one suggested by Buffet-Bataillon et al. [16]) to reduce the number of false positives. As a result, 69 species from each sample were considered for further analyses and discussion. These results are presented in Figure 3, with each heatmap corresponding to a different group of bacteria.

**Figure 4:**
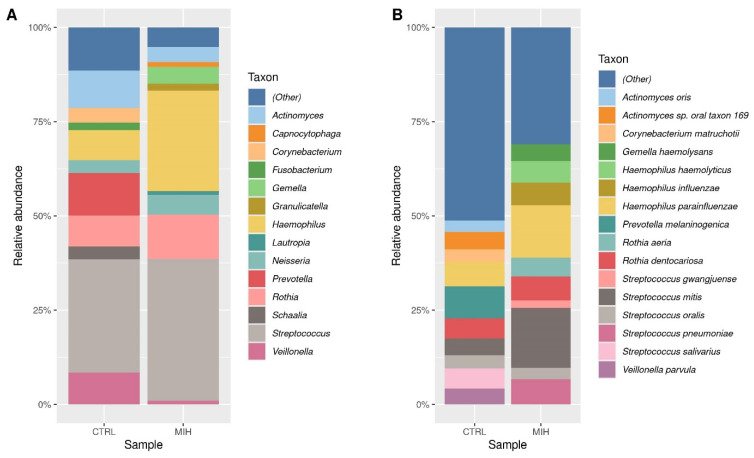
Relative abundances of bacteria in buccal epithelial samples from MIH-affected child and a healthy child at the genus (**A**) and species level (**B**).

A visual representation of the ten most abundant pathogens among the two children was created using relative abundance plots at the genus and species level. As shown in Figure 4A, the most abundant genera in the MIH-affected child were *Streptococcus* and *Haemophilus*. Other genera like *Gemella*, *Granulicatella*, *Capnocytophaga*, and *Lautropia*, which were among the top genera in the MIH sample, were present to a much lower extent in the healthy control sample. In contrast, we observed a higher number of bacteria from the genera *Corynebacterium*, *Fusobacterium*, *Prevotella*, and *Veilonella* in the healthy patient compared to the MIH-affected one.

A similar trend was found at the species level (shown in Figure 4B). Overall, the buccal microflora of the MIH-affected child presents slightly lower species diversity compared to that of the healthy child. We observed that only four species (namely, *Streptococcus mitis*, *Haemophilus parainfluenzae*, *Rothia dentocariosa*, and *Streptococcus oralis)* were similar in the top 10 groups of pathogens at the species level across the two individuals. *Gemella haemolysans* and *Haemophilus haemolyticus* were also found in the MIH-affected child, whereas in the healthy child they were present to a lower extent (and therefore included in the “Other” group).

The alpha diversity indices calculated to measure the richness and evenness of the microbiome in the two samples are presented in Table 2. Both Shannon and Simpson Diversity indices are slightly higher for the healthy child’s microbiome than for the MIH patient’s one, in accordance with the number of species present in these samples. The Berger–Parker index is higher for the MIH-affected child than for the healthy child, suggesting stronger dominance of certain groups, in particular *Streptococcus* and *Haemophilus*, which are more prevalent in the microbiome of the MIH-affected patient.

The Bray–Curtis dissimilarity index was used to measure beta diversity and equalled 0.72, which shows significant difference in the composition between these two microbiomes.

## 3. Discussion

The development and application of advanced molecular techniques have provided insight into the species diversity of the oral microbiome. To the best of our knowledge, our study is the first to use nanopore sequencing to compare the buccal microbiota in a child with MIH with a matched healthy child. Other researchers focused on the bacterial composition of supragingival dental plaque among MIH affected children using microscopy. The researchers also assessed the penetration of bacteria into the weakened enamel [17]. Scientists from Greece presented detection of a wide range of bacteria identified by their peptide sequences [18]. Our metagenomic analysis showed that the microorganisms that were more prevalent in the MIH patient’s sample were *Streptococcus mitis*, *Haemophilus parainfluenzae*, *Streptococcus pneumoniae*, *Rothia dentocariosa*, *Haemophilus influenzae*, *Haemophilus haemolyticus*, *Rothia aeria*, *Gemella haemolysans*, and *Streptococcus oralis*.

Some of the microorganisms detected in our study constitute commensal flora, while others have pathogenic potential. The genus *Rothia* is Gram-positive cocci that thrive in the oral environment, and includes several species like *R. aeria*, *R. mucilaginosa*, and *R. dentocariosa*. These microorganisms tend to be positively associated with oral health [19]. Our study has shown that *R. dentocariosa* and *R. aeria* predominate in patients with MIH, whereas *R. mucilaginosa* predominates in healthy individuals.

Other commensal bacteria, such as *Streptococcus mitis*, *S. oralis*, *S. sanguinis*, *Gemella haemolysans*, *Granulicatella elegans*, *Neisseria cinerea*, or *Neisseria sicca*, are predominant in the buccal mucosa microbiome of the patient with molar incisor hypomineralization (MIH) compared to the same bacteria in the age-matched healthy child. In contrast, the genetic material of *Neisseria subflava* was identified in higher amounts in the healthy individual.

Our study has shown that, among the selected Gram-positive cocci species, genetic material from bacteria with higher virulence potential, such as *Streptococcus pneumoniae*, predominates in the microbiome of the MIH patient. This bacterium commonly colonizes the nasopharynx but is also a significant cause of respiratory tract infections and invasive diseases [20]. Among the selected Gram-negative cocci species, a higher number of reads for *Neisseria meningitidis* was detected in the patient with MIH. This bacterium is part of the normal nasopharyngeal microbiome in healthy individuals, but it has a high virulence potential that can lead to septicemia and meningitis in vulnerable individuals [21]. A third clinically important pathogen, in addition to *Streptococcus pneumoniae* and *Neisseria meningitidis*, that is prevalent in patients with MIH is a Gram-negative rod, *Haemophilus influenzae.* This bacterium is a part of the commensal flora of the human upper respiratory tract and, due to its virulence factors, is also a pathogenic bacterium, causing both localized and invasive (septicemic) infections [22]. The significance of the co-occurrence of these three microorganisms in the buccal mucosal microbiome requires further study.

In our study, *Streptococcus mitis* was the predominant bacterium, and its genetic material was more than ten times higher in the patient with MIH compared to the healthy child. We also obtained interesting results regarding *Haemophilus* species, which were more numerous in the MIH patient. Perera et al. have shown that *H. parainfluenzae* is typically found accompanying the mitis group streptococci in vivo and is positively correlated with them based on microbiome data [23]. This team suggests that mitis group streptococci are likely the in vivo source of NAD for *H. parainfluenzae*, also inducing similar carbon utilization patterns in vitro to those seen in vivo. A study by Kilian et al. investigated the prevalence of *Haemophilus* bacteria in saliva and plaque [24]. The researchers found that *Haemophilus parainfluenzae* made up the majority of isolates from saliva, while *H. segnis* represented a significant proportion of the *Haemophilus* population in plaque. In our study, *Haemophilus parainfluenzae* showed the highest number of DNA fragments among the *Haemophilus* species detected, possibly due to the type of collected material.

Among the streptococci present on teeth are *Streptococcus mutans* and members of the *mitis* and *anginosus* groups. In contrast to the mucosal surface, *S. salivarius* is only a minor component of dental plaque. However, there is no doubt that *Streptococcus mutans* plays a key role in the initiation of tooth decay, while *Lactobacillus* spp. and *Actinomyces* spp. contribute to the exacerbation of cavities and the further progression of the disease [25,26,27]. An extensive study by Baker et al., which involved 23 children with caries and 24 children with healthy teeth, used saliva as the study material [28]. In the entire study group, *Rothia*, *Neisseria*, and *Haemophilus* spp. were associated with good dental health, while *Prevotella* spp., *Streptococcus mutans*, and human herpesvirus 4 (Epstein-Barr virus (EBV)) were more common in children with caries. In our study, however, we observed a decrease of *Streptococcus mutans* (not typically associated with the buccal mucosa) and *Prevotella* spp. in the patient with MIH compared to the healthy child. We suspect that this might be caused by the dominance of other pathogenic species in the MIH patient, which outcompeted these microbes.

*Gemella*, *Granulicatella*, *Capnocytophaga*, and *Lautropia* are part of the natural flora found in the oral cavity [29]. In our study, we observed the highest number of DNA fragments of *Gemella haemolysans*, *Granulicatella elegans*, *Capnocytophaga sputigena*, or *Lautropia mirabilis* in the patient with MIH in compared to the healthy individual. Kalpana et al. have shown positive correlation between *Leptotrichia*, which metabolises sucrose and its isomers via PTS (phosphotransferase system) to lactic acid in the absence of *Streptococcus mutans*. They have demonstrated that *Leptotrichia* could create an acidic environment for the growth of other acid bacteria such as *Capnocytophaga*, *Campylobacter*, *Gemella*, *Granulicatella*, or *Fusobacterium* [30]. As the researchers point out, the co-occurrence of these bacteria creates a strong network of complex microbial interactions.

The hard surface of the tooth is colonized by species of the genus *Actinomyces*. Our study has shown increased detection of *Actinomyces naeslundii* and of *A. viscosus* in the MIH-affected child compared to the healthy patient. These bacteria possess an enhanced capacity to attach to surfaces due to their specialized surface structures, known as type 1 and type 2 fimbriae. The proportions of *Streptococcus mutans* and *Actinomyces viscosus* are significantly higher in cases of confirmed enamel demineralization. The isolation of *A. viscosus* from early carious enamel lesions is known to indicate a high risk of cavity formation [31,32,33]. Analysis of the microbial salivary proteome by Pappa et al. showed lower microbial species diversity in patients with MIH. The authors highlight the dysbiotic environment present in MIH pathology [18]. Their research revealed deregulation of proteins involved in antioxidant defence, immune response, acute inflammation, and complement activation in MIH-affected children. The deregulation of the immune system and bacterial defence response, combined with the porosity of the affected enamel, alters the host-microbe balance and leads to oral dysbiosis. In agreement with that study, we also observed a lower microbial species diversity in the patient with MIH with respect to that of healthy child, as presented by alpha and beta diversity indices. The Bray–Curtis dissimilarity index assesses ecological beta diversity of detected bacteria. The result (0.72) indicates strong differences in species composition between children. The reduction in the diversity of the buccal microflora in a patient with MIH was also supported by other indexes, Shannon and Simpson Diversity Indexes, which measure alpha diversity. This was further supported by the Berger–Parker Diversity Index, indicating clear dominance of certain taxa above others (further reduction in diversity) in the MIH-affected child.

The main limitation of the study was the number of analysed patients. We decided to focus our analyses on a single measurement among a MIH-affected child and a matching healthy child. Although this enabled us to explore the differences in the buccal microbiome between the two patients, a follow-up study on a larger cohort is needed to generalise our findings and draw more comprehensive conclusions. Likewise, in future experiments, we plan to explore the ONT adaptive sampling method to increase the overall microbial resolution by depleting off-target reads that would map to the host genome.

## 4. Materials and Methods

### 4.1. Ethical Considerations

The Medical Ethics Committee of the Medical University of Silesia in Katowice, Poland approved the study protocol (PCN/CBN/0022/KB1/108/IV/19/20/21/22, PCN/CBN/0052/KB1/145/21/22).

After providing parents or legal guardians with information regarding the study’s objectives, we obtained written informed consent for the study.

### 4.2. Clinical Examination

The criteria for the identification of MIH were established by the European Academy of Paediatric Dentistry [3]. Dental professionals were familiarized with the EAPD guidelines and trained to avoid misdiagnosis.

Dentists examined 90 children during dental visits in the Developmental Age Clinic of the University Dental Center of the Silesian Medical University in Katowice, Poland. There were 21 girls and 27 boys in the study group and 24 girls and 18 boys in the control group.

During dental visits, we conducted a detailed clinical questionnaire with emphasis on potential aetiological factors. Children underwent a medical interview and a dental examination to assess their general and dental health status. The examination of all children showed no congenital defects, cranio-mandibular disorders, or genetic disorders in the mouth and teeth. There were no signs of infection or inflammation in the oral tissues. Children were in good general health with no symptoms of common infectious diseases. They were not taking any medications, either permanently or temporarily. Negative dental and general health status were primary selection criteria. All children were included in the preliminary study and control groups. Then, buccal mucosal epithelial cell samples were then collected from all participants using sterilised cotton swabs.

The secondary criteria for the selection of the sample of the children selected for the study were the following: a complete clinical history with special attention to the aetiological factors of MIH, age and gender concordance, and high DNA content in the samples after DNA isolation. Two children who met the secondary selection criteria were included in the study, including one child diagnosed with MIH and one without MIH (healthy child). The clinical characteristics of these children are presented in Table 3.

### 4.3. Sample Collection

The tissue sampling protocol involved collecting buccal mucosa epithelial cell samples using sterilized cotton swabs (Dentalab, Palma, Spain) to scrape off the exfoliated cells of the buccal mucosa from the right and left cheeks of the patients. For further laboratory testing, the collected material was protected from degradation by storage at sub-zero temperatures.

### 4.4. DNA Isolation

DNA from buccal mucosal epithelial cells was isolated using the GeneMATRIX Swab-Extract DNA Purification Kit (Eurx, Gdańsk, Poland, #E3530-02) according to the manufacturer’s instructions. The concentration and purity of the DNA was determined via spectrophotometry using a NanoPhotometer Pearl UV/Vis spectrophotometer (Implen, Munich, Germany). After isolation, samples were stored at −20 °C and transported for further analyses on dry ice, and then stored at −80 °C until further processing.

### 4.5. ONT Sequencing

In addition to spectrophotometric quality control (QC), the extracts were QC’d prior to sequencing using a Qubit 4 fluorometer (ThermoFisher Scientific, Waltham, MA, USA) and a dsDNA HS kit (ThermoFisher Scientific, Waltham, MA, USA, #Q33230) according to the attached protocol. Genomic tape and reagents (Agilent, Santa Clara, CA, USA, #5067-5365; #5067-5366) were used to measure the DNA fragment length and DIN values of the extracted DNA via the Tapestation 4200 instrument (Agilent, Santa Clara, CA, USA) according to the protocol.

Libraries were prepared following the “Native barcoding genomic DNA” protocol, using a Ligation kit and a Native Barcoding Expansion kit 13–24 from Oxford Nanopore Technologies (Oxford, UK, #SQK-LSK109 and #EXP-NBD114, respectively).

Samples were sequenced in triplicate using PromethION R9 flow cells on a P2solo device using MinKNOW software version 24.02.16. (Oxford Nanopore Technologies, Oxford, UK). Each run/flow cell involved healthy control (child without MIH) and the corresponding MIH sample, barcoded with separate barcodes (23 and 24, respectiely). Each run lasted 72 h.

### 4.6. Bioinformatic Data Analysis

ONT raw sequencing data underwent an initial QC step to inspect read distribution and quality using NanoPlot (v1.43.0) [34] with options “--huge --no_static --N50”, followed by a trimming step with Porechop (v0.2.4) [35] to remove ONT adapters and retain any split reads longer than 100 bp using option “--min_split_read_size 100”, and by a filtering step with NanoFilt (v2.8.0) [36] to discard low-quality reads using option “--quality 9”. Next, surviving reads were used as input for dustmasker (v1.0.0) [37] with options “-hard_masking -outfmt” to mask any low-complexity regions, and were subsequently mapped to the human reference genome (assembly GRCh38.p12) with minimap2 (v2.28-r1209) [38] using options “-x map-ont -a --secondary = no” in order to perform a host removal step. Non-human reads were then used in Kraken2 (v2.1.3) [39] with options “--memory-mapping --confidence 0.05 --minimum-hit-groups 3” to perform taxonomic classification, and results were visualised using Krona (v2.8.1) [40]. Finally, Bracken (v3.0) [41] was used, with default settings, to estimate relative abundances at the species, genus, and family level. Alpha- and beta-diversity indices were calculated using KrakenTools (v1.2) [42].

## 5. Conclusions

Our results show differences in buccal microbiome diversity and composition between MIH affected and healthy individuals, with higher microbial diversity found in a healthy child. The buccal microbiome can potentially act as a reservoir of pathogens and therefore contribute to further progression of oral and general diseases. This study provides data as a starting point for further research to be conducted on a larger study group, which will also expand the scope to include samples from teeth and gingiva. This should help answer the questions raised by this study and lead to a practical application of these discoveries in a clinical setting.

## Figures and Tables

**Table 1:** Sequencing and filtering metrics for the control and MIH samples. Values are expressed in the following units of measurement, shown between parentheses: “n” (=counts), “bp” (=base pairs), and “Q” (Phred score).

Bioinformatic Analysis Workflow Step	Control Sample	MIH Sample
Initial read count (n)	31,152,066	25,079,108
Mean read length (bp)	737.3	1013.9
Longest read length (bp)	353,802	280,616
N50 read length (bp)	1247	1532
Average read quality before quality filtering (Q)	11.7	11.9
Basepairs removed during ONT adapter trimming (n)	131,461,225	56,179,806
Reads surviving quality filtering (n)	29,294,073	24,098,175
Average read quality after quality filtering (Q)	12.2	12.2
Basepairs replaced by Ns during LCR masking (n)	1,314,261,895	1,339,951,641
Reads mapping to the host reference genome (n)	27,531,354	22,563,726
Host-removed reads used for Kraken2 and Bracken analyses (n)	1,762,719	1,534,449

**Table 2:** Alpha diversity indices healthy child and MIH patient.

Patient	Shannon Diversity Index	Simpson Diversity Index	Berger-Parker Diversity Index
Healthy child	4.10	0.97	0.09
MIH-affected child	3.37	0.93	0.16

**Table 3:** The clinical profiles of selected patients.

		MIH	Control
Gender		female	female
Age		7	7
MIH TNI Index [5]		4b	0
EAPD Criteria [3]	Demarcated opacities	Present	No
	Posteruptive enamel breakdown	Present	No
	Atipical restorations	No	No
	Extractions of molars due to MIH	No	No
	Failure of eruption of molar or an incisor	Permanent upper lateral incisors	Permanent right upper lateral incisor
Miscarriage		No	No
Complications during labour		Yes	No
Smoking cigarettes during pregnancy		No	No
Alcohol consumption during pregnancy		No	No
Folic acid supplementation		Yes	Yes
Vitamins supplementation during pregnancy		No	Yes
Drugs during pregnancy		Yes	Yes
Diseases during pregnancy		Yes	Yes
Type of labour		Natural childbirth	Natural childbirth
Varicella infection under the age of 3		No	No
Ear infection under the age of 3		No	No
Pneumonia under the age of 3		No	No
Asthma under the age of 3		No	No
Bronchitis under the age of 3		No	Yes
Fever episodes > 39 °C under the age of 3		No	No
Breast-feeding		No	Yes

## Data Availability

The raw data supporting the conclusions of this article will be made available by the authors on request.

## Supplementary Materials

The following supporting information can be downloaded at: https://www.mdpi.com/article/10.3390/ijms252313143/s1.
