# Supplementary material for: Metagenomic Analysis of the Buccal Microbiome by Nanopore Sequencing Reveals Structural Differences in the Microbiome of a Patient with Molar Incisor Hypomineralization (MIH) Compared to a Healthy Child—Case Study
Source: Int J Mol Sci. 2024 Dec 6;25(23):13143. doi: 10.3390/ijms252313143 (PMC11642311; doi:10.3390/ijms252313143)
Supplement: Supplementary file 1 [file ijms-25-13143-s001.zip › Figure S1. Epithelium_CTRL_krona_plot.html]

Javascript must be enabled to view this page.

members
magnitude
magnitudeUnassigned
count
unassigned
taxon
rank

Epithelium\_CTRL\_non\_human\_reads\_kraken\_out

node0.members.0.js
2405
1762719

24967
node1.members.0.js
2759
superkingdom
856463

12
2698737
clade

33630
11
clade

phylum
5794
11

1
422676
class

order
5863
1

1
32594
family

5864
1
genus

species
node9.members.0.js
5866
1

1280412
10
class

subclass
10
5796

order
75739
10

suborder
10
423054

10
5809
family

genus
10
5810

species
5811
10

node17.members.0.js
strain
508771
10

33634
1
clade

2696291
1
clade

phylum
2836
1

class
1
33836

33846
1
subclass

order
33847
1

29202
1
family

genus
1
35127

species
1
35128

node27.members.0.js
strain
296543
1

2611352
2
clade

phylum
33682
2

5653
2
class

subclass
2
2704647

order
2704949
2

5654
2
family

1286322
2
subfamily

genus
5658
2

2
38568
subgenus

2
38582
species group

5665
2
species

2
929439
node39.members.0.js
strain

clade
831482
node40.members.0.js
24
33154

kingdom
28
4751

subkingdom
451864
28

phylum
5204
19

452284
19
subphylum

1538075
19
class

19
162474
order

family
19
742845

genus
19
55193

node49.members.0.js
species
76775
19

phylum
9
4890

716545
node51.members.0.js
1
9
clade

147537
2
subphylum

4891
2
class

2
4892
order

4893
1
family

1
4930
genus

1
4932
species

1
559292
node58.members.0.js
strain

clade
2916678
1

1
766764
family

genus
1
4958

species
4959
1

node63.members.0.js
strain
284592
1

147538
6
subphylum

clade
6
716546

147545
5
class

subclass
5
451871

5
5042
order

family
5
1131492

genus
5073
1

species
node71.members.0.js
1
69781

genus
5052
4

4
2720874
subgenus

species
node74.members.0.js
182096
4

clade
715989
1

class
1
147550

subclass
222543
1

1
5125
order

1
34397
family

genus
124426
1

1
1159556
species
node81.members.0.js

831430
33208
kingdom

6072
831430
clade

clade
831430
33213

33511
831430
clade

831430
7711
phylum

subphylum
831430
89593

7742
831430
clade

clade
7776
831430

clade
831430
117570

clade
831430
117571

831430
8287
superclass

clade
1338369
831430

clade
831430
32523

32524
831430
clade

40674
831430
class

831430
32525
clade

9347
831430
clade

clade
831430
1437010

superorder
314146
831430

order
831430
9443

831430
376913
suborder

infraorder
314293
831430

parvorder
831430
9526

superfamily
831430
314295

831430
9604
family

207598
831430
subfamily

genus
831430
9605

9606
831430
species
node109.members.0.js

node110.members.0.js
627698

10239
97
superkingdom

96
2731341
clade

kingdom
2731360
96

65
2731618
phylum

2731619
65
class

no rank
51
node116.members.0.js
2
2788787

species
node117.members.0.js
1566990
1

12402
6
node118.members.0.js
species

node119.members.0.js
species
42
644007

6
2842328
family

node121.members.0.js
3
1982583
genus
6

species
1982584
3

no rank
node123.members.0.js
10747
3

3044455
node124.members.0.js
1
8
family

no rank
5
3094845

species
node126.members.0.js
5
537874

2
genus
1623304
node127.members.0.js
1

3060358
1
species

node129.members.0.js
no rank
1971421
1

31
2731361
phylum

class
31
2731363

548681
31
order

3044472
31
family

subfamily
30
10374

genus
30
1
node135.members.0.js
10375

3050299
29
species

29
no rank
10376
node137.members.0.js
24

12509
5
node138.members.0.js
no rank

1
10357
subfamily

genus
40272
1

3050298
1
species

1
10372
no rank
node142.members.0.js

family
1
687329

genus
node144.members.0.js
1
687331

1943
node145.members.0.js
2
superkingdom
276055

29547
2084
phylum

2084
3031852
class

order
2084
1
node148.members.0.js
213849

72294
2083
family

2083
genus
194
54
node150.members.0.js

204
109
node151.members.0.js
species

node152.members.0.js
species
372
824

12
203
node153.members.0.js
species

200
3
species
node154.members.0.js

species
1531
node155.members.0.js
1483
199

48
360104
node156.members.0.js
strain

2593542
2
no rank

1032243
1
species
node158.members.0.js

species
node159.members.0.js
1
2517362

phylum
1
57723

204432
1
class

1
204433
order

family
1
204434

genus
1
33973

33075
1
species

node166.members.0.js
strain
240015
1

1
200940
phylum
node167.members.0.js

phylum
10360
32066

203490
10360
class

order
10360
node170.members.0.js
29
203491

family
4854
8
node171.members.0.js
1129771

14
2755140
genus

14
157692
species
node173.members.0.js

4832
genus
32067
node174.members.0.js
1356

species
274
40542

274
523794
strain
node176.members.0.js

species
node177.members.0.js
612
157687

157691
29
species
node178.members.0.js

1071
no rank
2633022
34
node179.members.0.js

node180.members.0.js
species
3058373
140

node181.members.0.js
species
27
712368

species
node182.members.0.js
712362
20

712357
819
node183.members.0.js
species

node184.members.0.js
species
17
1785996

species
node185.members.0.js
14
712361

species
node186.members.0.js
754
554406

species
node187.members.0.js
423
157688

species
node188.members.0.js
109328
313

5477
family
203492
node189.members.0.js
1

5476
genus
848
2863
node190.members.0.js

76857
1070
node191.members.0.js
species

851
node192.members.0.js
38
39
species

1
76856
subspecies

node194.members.0.js
strain
525283
1

2663009
1036
node195.members.0.js
species

species
9
8
node196.members.0.js
155615

1
1307427
strain
node197.members.0.js

285729
7
node198.members.0.js
species

species
node199.members.0.js
401
76859

node200.members.0.js
species
860
37

1
859
species
node201.members.0.js

13
species
1583098
11
node202.members.0.js

1307443
1
strain
node203.members.0.js

1
1307442
node204.members.0.js
strain

222
node205.members.0.js
1224
phylum
34967

28211
6
node206.members.0.js
69
class

order
204458
20

76892
node208.members.0.js
2
20
family

41275
node209.members.0.js
9
18
genus

no rank
3
node210.members.0.js
1
2622653

1
2579977
species
node211.members.0.js

1
3068314
node212.members.0.js
species

node213.members.0.js
species
2
41276

1
114552
no rank

1
213418
species
node215.members.0.js

species
node216.members.0.js
74329
3

17
order
356
1
node217.members.0.js

4
family
41294
1
node218.members.0.js

2
node219.members.0.js
374
genus
3

node220.members.0.js
no rank
2631580
1

family
1
69277

1
68287
genus

2725666
1
species
node223.members.0.js

1
255475
family

1
414371
genus

2615206
1
no rank

1
2816454
node227.members.0.js
species

family
82115
1

no rank
227290
1

node230.members.0.js
genus
357
1

family
119045
6

node232.members.0.js
genus
1
2282523

407
3
node233.members.0.js
5
genus

1
2615210
no rank

2603276
1
species
node235.members.0.js

410
1
species
node236.members.0.js

family
1
2831106

genus
1
3064792

1
2909283
species
node239.members.0.js

2
2831100
family

85413
node241.members.0.js
1
2
genus

node242.members.0.js
species
3068632
1

order
2
204441

2
433
family

2
125216
genus
node245.members.0.js

204455
node246.members.0.js
1
12
order

11
family
31989
node247.members.0.js
1

node248.members.0.js
4
265
genus
10

species
node249.members.0.js
1
2259340

3
59779
node250.members.0.js
species

2688777
1
no rank

node252.members.0.js
species
1
2500532

1
2903900
species
node253.members.0.js

204457
node254.members.0.js
4
12
order

1
node255.members.0.js
41297
family
7

165695
3
genus

no rank
1
2611147

species
node258.members.0.js
1
627192

2
13690
species
node259.members.0.js

3
genus
13687
1
node260.members.0.js

no rank
1
196159

1961362
1
species
node262.members.0.js

563996
1
species
node263.members.0.js

1
335929
family

1
2800686
genus

1
2338327
node266.members.0.js
species

1236
155
node267.members.0.js
23252
class

21842
135625
order

family
21842
node269.members.0.js
484
712

genus
10
node270.members.0.js
6
713

species
1
718

202948
1
node272.members.0.js
subspecies

node273.members.0.js
species
2
51049

node274.members.0.js
species
716
1

2019
node275.members.0.js
724
genus
21074

16791
species
729
node276.members.0.js
16573

862965
218
node277.members.0.js
strain

species
node278.members.0.js
1
197575

species
node279.members.0.js
98
735

node280.members.0.js
species
249188
15

327
node281.members.0.js
727
species
401

1295140
1
strain
node282.members.0.js

strain
node283.members.0.js
1232659
2

strain
node284.members.0.js
862964
1

70
725
biotype
node285.members.0.js

730
1
node286.members.0.js
species

2609962
320
no rank

node288.members.0.js
species
320
712310

species
node289.members.0.js
1428
726

genus
1960084
1

node291.members.0.js
species
2778911
1

1
697331
genus

1
157673
species

221988
1
strain
node294.members.0.js

745
1
node295.members.0.js
7
genus

747
6
species
node296.members.0.js

292486
1
node297.members.0.js
genus

genus
3
2094023

2
738
species
node299.members.0.js

1
2629322
no rank

species
node301.members.0.js
2030797
1

node302.members.0.js
39
416916
genus
254

species
50
41
node303.members.0.js
732

985008
5
node304.members.0.js
strain

634176
4
node305.members.0.js
strain

node306.members.0.js
species
9
739

no rank
146
node307.members.0.js
22
2639383

species
node308.members.0.js
6
2866570

2820817
94
node309.members.0.js
species

node310.members.0.js
species
24
712150

10
714
node311.members.0.js
species

genus
7
node312.members.0.js
3
75984

node313.members.0.js
species
2
75985

species
node314.members.0.js
2770636
1

85402
1
species
node315.members.0.js

135622
3
order

family
2
267890

22
2
genus

species
70863
1

node320.members.0.js
strain
211586
1

271098
1
species

node322.members.0.js
strain
1
458817

267891
1
family

genus
58050
1

no rank
2637987
1

1
2746230
node326.members.0.js
species

1
135619
order

28256
1
family

1
2745
genus
node329.members.0.js

135624
1
order

84642
1
family

1
642
node332.members.0.js
genus

91347
node333.members.0.js
9
29
order

1903411
1
family

1
613
node335.members.0.js
genus

family
1
1903410

1
204037
node337.members.0.js
genus

543
9
node338.members.0.js
18
family

node339.members.0.js
genus
544
1

3
no rank
2890311
node340.members.0.js
1

genus
2
570

2
1134687
species
node342.members.0.js

genus
5
node343.members.0.js
1
561

species
node344.members.0.js
1
564

562
3
species
node345.members.0.js

order
1
135623

641
1
family
node347.members.0.js

2887326
212
order

212
family
468
node349.members.0.js
1

genus
135
21
node350.members.0.js
475

no rank
3
2685852

3
3076087
species
node352.members.0.js

34062
111
node353.members.0.js
species

497
1
genus

no rank
196806
1

species
node356.members.0.js
3064892
1

36
node357.members.0.js
469
genus
75

909768
1
species group

1
470
node359.members.0.js
species

node360.members.0.js
species
1
202956

1
1776741
species
node361.members.0.js

node362.members.0.js
species
29430
1

9
28090
node363.members.0.js
species

4
108980
node364.members.0.js
species

3
node365.members.0.js
196816
no rank
9

species
node366.members.0.js
1
3003392

node367.members.0.js
species
4
3045147

2743575
1
node368.members.0.js
species

12
species
40214
node369.members.0.js
11

strain
node370.members.0.js
1242245
1

node371.members.0.js
species
1
1776742

135615
265
order

265
868
family

node374.members.0.js
20
2717
genus
265

species
node375.members.0.js
222
2718

no rank
23
2648856

2866573
23
species
node377.members.0.js

order
727
node378.members.0.js
1
135614

32033
10
node379.members.0.js
726
family

338
node380.members.0.js
59
711
genus

339
642
node381.members.0.js
652
species

1
92826
node382.members.0.js
no rank

9
no rank
340
4
node383.members.0.js

node384.members.0.js
strain
1281284
1

1358002
4
node385.members.0.js
strain

40323
1
node386.members.0.js
genus

83618
3
genus

128785
1
node388.members.0.js
species

1
314722
node389.members.0.js
species

1
266062
species
node390.members.0.js

genus
83614
1

no rank
1
2629088

species
node393.members.0.js
2799326
1

72274
16
order

family
16
node395.members.0.js
1
135621

3
2901164
genus

3
136846
species group

species subgroup
3
578833

species
node399.members.0.js
3
316

286
5
node400.members.0.js
12
genus

species group
136845
4

node402.members.0.js
species
303
4

136841
1
species group

287
1
node404.members.0.js
species

species group
1
136843

129817
1
node406.members.0.js
species

1
1691904
node407.members.0.js
species

class
11424
40
node408.members.0.js
28216

597
order
80840
node409.members.0.js
20

family
1
75682

1
202907
genus

1
158899
species
node412.members.0.js

4
node413.members.0.js
80864
family
40

genus
34
2
node414.members.0.js
219181

no rank
32
2645081

1658672
32
species
node416.members.0.js

3051137
1
genus

80867
1
species
node418.members.0.js

1
12916
genus

1
2684926
node420.members.0.js
no rank

506
node421.members.0.js
1
2
family

1
222
node422.members.0.js
genus

family
534
node423.members.0.js
3
119060

genus
32008
9

87882
9
species group

9
87883
species
node426.members.0.js

47670
522
genus

522
47671
node428.members.0.js
species

10787
order
206351
node429.members.0.js
13

family
10774
node430.members.0.js
1001
481

482
node431.members.0.js
5360
8597
genus

species
20
18
node432.members.0.js
486

strain
node433.members.0.js
2
489653

487
58
node434.members.0.js
66
species

serogroup
491
1

1
630588
node436.members.0.js
strain

4
662598
strain
node437.members.0.js

serogroup
135720
3

node439.members.0.js
strain
1
604162

2
374833
strain
node440.members.0.js

151
species
495
node441.members.0.js
124

node442.members.0.js
subspecies
88719
27

node443.members.0.js
species
483
264

493
2
species
node444.members.0.js

1
194197
species
node445.members.0.js

species
496
212

997348
212
strain
node447.members.0.js

42
484
species
node448.members.0.js

species
node449.members.0.js
1
1853276

1
2995413
node450.members.0.js
species

node451.members.0.js
species
1
326522

node452.members.0.js
species
28449
894

99
267212
node453.members.0.js
species

species
531
node454.members.0.js
530
490

strain
node455.members.0.js
547045
1

species
node456.members.0.js
10
485

species
node457.members.0.js
1
28091

1
2709396
node458.members.0.js
species

2623750
node459.members.0.js
71
597
no rank

64
3077590
node460.members.0.js
species

24
2937985
species
node461.members.0.js

node462.members.0.js
species
155
2972775

species
node463.members.0.js
31
655307

species
node464.members.0.js
2
2912675

species
191
641148

node466.members.0.js
strain
641149
191

59
1871109
species
node467.members.0.js

node468.members.0.js
species
2
1853278

species
node469.members.0.js
326523
1

species
node470.members.0.js
2666100
1

281
488
node471.members.0.js
species

node472.members.0.js
species
33053
58

2944815
1
genus

2917790
1
node474.members.0.js
species

71
4
genus

species
node476.members.0.js
72
4

201
genus
538
node477.members.0.js
20

12
2528037
species
node478.members.0.js

species
node479.members.0.js
169
539

genus
334107
1

species
node481.members.0.js
153493
1

genus
856
node482.members.0.js
10
32257

species
node483.members.0.js
505
700

1
1522312
species
node484.members.0.js

145
502
species
node485.members.0.js

genus
212742
113

113
1056807
species
node487.members.0.js

phylum
1
200783

class
187857
1

order
1
32069

family
64898
1

939
1
genus

no rank
2647298
1

species
node494.members.0.js
1
1632019

1783272
node495.members.0.js
189
186640
clade

3
544448
phylum

2790996
3
order

3
2895623
family

genus
2767358
1

species
node500.members.0.js
48003
1

2
2923352
genus

species
node502.members.0.js
2
29562

114426
phylum
1239
node503.members.0.js
262

class
161
1737404

order
161
1737405

1570339
161
family

158
543311
genus

158
33033
node508.members.0.js
species

1
162289
genus

node510.members.0.js
species
1
54005

genus
150022
2

node512.members.0.js
species
1260
2

157
526524
class

526525
157
order

128827
157
family

genus
154
123375

154
102148
species
node517.members.0.js

3
433334
no rank

1522
3
node519.members.0.js
species

186801
node520.members.0.js
33
1039
class

order
68295
1

186814
1
family

1
28895
genus

2622527
1
no rank

1
1550240
node525.members.0.js
species

order
399
3082720

396
3030910
family

86331
node528.members.0.js
26
396
genus

species
node529.members.0.js
114527
281

node530.members.0.js
species
13
86332

114528
76
species
node531.members.0.js

3
186804
family

1
1257
genus

species
node534.members.0.js
3003601
1

genus
1870884
2

species
node536.members.0.js
2
1496

3085636
583
order

583
family
186803
node538.members.0.js
22

genus
522
88
node539.members.0.js
1164882

node540.members.0.js
species
88
617123

species
node541.members.0.js
346
2490855

node542.members.0.js
genus
1
572511

1506553
1
genus

1
1871021
species
node544.members.0.js

1
841
genus

node546.members.0.js
species
166486
1

genus
6
1766253

species
6
5
node548.members.0.js
39491

node549.members.0.js
strain
657318
1

genus
2316020
2

species
node551.members.0.js
33038
2

28
1213720
genus

no rank
28
2643635

species
node554.members.0.js
3059030
28

186802
node555.members.0.js
1
23
order

1
2937911
family

genus
278993
1

species
1
863643

635013
1
node559.members.0.js
strain

1
538999
no rank

genus
node561.members.0.js
1
1686313

31979
1
node562.members.0.js
13
family

genus
11
node563.members.0.js
2
1485

node564.members.0.js
species
1
36845

node565.members.0.js
species
1491
8

1
44258
genus

1
2629145
no rank

node568.members.0.js
species
3023090
1

7
family
216572
1
node569.members.0.js

2
genus
216851
1
node570.members.0.js

1
2646395
node571.members.0.js
no rank

1
236752
genus

species
node573.members.0.js
1
236753

genus
2834348
3

3
2834349
no rank

node576.members.0.js
species
3
2994078

class
23477
9
node577.members.0.js
909932

425
order
909929
node578.members.0.js
1

1843491
424
family

2
158846
genus

node581.members.0.js
species
158847
2

genus
422
node582.members.0.js
81
970

94
species
69823
node583.members.0.js
80

546271
14
node584.members.0.js
strain

species
node585.members.0.js
14
2754044

species
node586.members.0.js
22
135079

211
no rank
2637378
node587.members.0.js
83

1884263
34
node588.members.0.js
species

species
node589.members.0.js
7
712528

species
node590.members.0.js
713030
44

43
712538
node591.members.0.js
species

order
23043
1843489

31977
1
node593.members.0.js
23043
family

39948
node594.members.0.js
5
7
genus

species
node595.members.0.js
2
39950

2
genus
906
1
node596.members.0.js

node597.members.0.js
species
1
1232428

29465
node598.members.0.js
3699
23033
genus

species
node599.members.0.js
3512
423477

node600.members.0.js
species
2547
39777

no rank
2630086
1058

species
node602.members.0.js
1058
2682455

2682456
1077
species
node603.members.0.js

10634
species
29466
10115
node604.members.0.js

strain
node605.members.0.js
519
1316254

481
39778
node606.members.0.js
species

25
248315
species
node607.members.0.js

class
89330
node608.members.0.js
299
91061

node609.members.0.js
379
186826
order
84685

family
504
186827

node611.members.0.js
2
1375
genus
6

node612.members.0.js
species
1377
3

node613.members.0.js
species
51665
1

46123
498
genus

498
species
46125
node615.members.0.js
326

172
592010
strain
node616.members.0.js

81852
1
node617.members.0.js
41
family

node618.members.0.js
8
1350
genus
40

1354
2
node619.members.0.js
species

no rank
2608891
2

1
2057791
node621.members.0.js
species

1
2230877
node622.members.0.js
species

1
1351
node623.members.0.js
species

node624.members.0.js
species
27
1352

family
80862
node625.members.0.js
53
1300

genus
15
2
node626.members.0.js
1357

8
species
1359
5
node627.members.0.js

2816960
3
subspecies

node629.members.0.js
strain
1449093
3

node630.members.0.js
species
1358
5

genus
80794
39304
node631.members.0.js
1301

species
4577
node632.members.0.js
4100
1303

1458253
104
node633.members.0.js
subspecies

subspecies
node634.members.0.js
1077464
81

205
1891914
subspecies
node635.members.0.js

strain
node636.members.0.js
87
927666

node637.members.0.js
species
2382163
39

1335
3
node638.members.0.js
species

species
4469
node639.members.0.js
4208
1304

41
1200793
node640.members.0.js
strain

347253
72
node641.members.0.js
strain

99
1048332
node642.members.0.js
strain

strain
node643.members.0.js
1046629
43

strain
node644.members.0.js
6
1074494

species
node645.members.0.js
29
2490633

species
node646.members.0.js
2993430
382

node647.members.0.js
species
1310
2

species
node648.members.0.js
361101
2

species
node649.members.0.js
2
2686210

33040
8
species
node650.members.0.js

2
1341
node651.members.0.js
species

species
2750
node652.members.0.js
2099
1318

node653.members.0.js
strain
1114965
324

760570
327
node654.members.0.js
strain

34
1234680
node655.members.0.js
species

8
1501662
species
node656.members.0.js

6848
species
28037
node657.members.0.js
6378

strain
node658.members.0.js
470
365659

node659.members.0.js
3217
1305
species
3579

strain
node660.members.0.js
388919
362

82348
5
node661.members.0.js
species

123
684066
node662.members.0.js
species

79
1156431
node663.members.0.js
species

node664.members.0.js
species
4
1917441

285
113107
node665.members.0.js
species

node666.members.0.js
species
1
315405

2
29389
node667.members.0.js
species

node668.members.0.js
1218
1309
species
1235

strain
node669.members.0.js
3
1155071

strain
node670.members.0.js
1
1441364

1225197
6
strain
node671.members.0.js

511691
4
strain
node672.members.0.js

3
1198676
node673.members.0.js
strain

4
1111760
node674.members.0.js
species

node675.members.0.js
177
1308
species
184

strain
node676.members.0.js
1433289
1

strain
node677.members.0.js
1436725
2

node678.members.0.js
strain
1435981
1

1435974
1
strain
node679.members.0.js

node680.members.0.js
strain
1051074
2

species
node681.members.0.js
2819619
443

1
1348
node682.members.0.js
species

1307
18
node683.members.0.js
species

43
1343
species
node684.members.0.js

species
node685.members.0.js
1433513
803

species
node686.members.0.js
1
102886

species
node687.members.0.js
1
1814128

2
2173853
species
node688.members.0.js

no rank
10203
3059
node689.members.0.js
2608887

2975324
228
node690.members.0.js
species

node691.members.0.js
species
1109
3077584

794
2972784
species
node692.members.0.js

3077723
171
species
node693.members.0.js

species
node694.members.0.js
75
1759399

species
node695.members.0.js
3098076
283

species
node696.members.0.js
2598453
357

node697.members.0.js
species
2763068
6

node698.members.0.js
species
109
712624

662
2759692
node699.members.0.js
species

45
2954545
node700.members.0.js
species

species
node701.members.0.js
2975349
102

species
node702.members.0.js
1540
712633

node703.members.0.js
species
2598457
24

node704.members.0.js
species
712623
628

species
node705.members.0.js
83
2576376

83
3038077
node706.members.0.js
species

species
node707.members.0.js
321
2610896

species
node708.members.0.js
52
2954513

1839799
132
species
node709.members.0.js

node710.members.0.js
species
235
3098075

node711.members.0.js
species
101
1902136

2420310
4
node712.members.0.js
species

1329
1
species
node713.members.0.js

16
59310
node714.members.0.js
species

34
1311
species
node715.members.0.js

species
322
5
node716.members.0.js
257758

strain
node717.members.0.js
317
1054460

14
119603
species group

species
8
1336

subspecies
8
node720.members.0.js
6
40041

2
1051072
strain
node721.members.0.js

species
6
node722.members.0.js
3
1334

node723.members.0.js
subspecies
119602
1

node724.members.0.js
subspecies
2
99822

species
2070
node725.members.0.js
2050
1313

3
516950
strain
node726.members.0.js

1
697283
strain
node727.members.0.js

node728.members.0.js
strain
1
869215

1
869216
node729.members.0.js
strain

strain
node730.members.0.js
1130804
3

3
488222
strain
node731.members.0.js

487214
5
node732.members.0.js
strain

strain
node733.members.0.js
3
1159083

species group
811
88
node734.members.0.js
671232

1328
173
node735.members.0.js
175
species

node736.members.0.js
strain
862970
1

1
1272910
subspecies

1
1353243
node738.members.0.js
strain

76860
25
species
node739.members.0.js

node740.members.0.js
488
1338
species
523

strain
node741.members.0.js
862966
10

25
862967
node742.members.0.js
strain

node743.members.0.js
species
1302
1633

45634
62
node744.members.0.js
150
species

strain
node745.members.0.js
889201
87

strain
node746.members.0.js
1
1302863

species
node747.members.0.js
18
1314

1345
1
node748.members.0.js
species

2781599
2
species
node749.members.0.js

247
68892
node750.members.0.js
species

93
family
33958
10
node751.members.0.js

1
2767885
genus

species
node753.members.0.js
1
1599

2742598
4
genus

1613
4
node755.members.0.js
species

1
2767893
genus

1
1581
species
node757.members.0.js

1243
3
node758.members.0.js
4
genus

1252
1
node759.members.0.js
species

6
2767887
genus

1624
5
node761.members.0.js
species

species
node762.members.0.js
1
1622

genus
49
node763.members.0.js
3
2759736

4
47715
node764.members.0.js
species

1597
42
node765.members.0.js
species

genus
2
2767842

species
node767.members.0.js
1
1589

species
node768.members.0.js
1590
1

1
2767888
genus

species
468911
1

node771.members.0.js
strain
1
1423759

13
genus
1578
8
node772.members.0.js

species
node773.members.0.js
47770
1

species
2107999
1

node775.members.0.js
strain
525326
1

1596
2
node776.members.0.js
species

species
1
1584

1
249265
node778.members.0.js
subspecies

genus
2
46255

2
1631871
species
node780.members.0.js

2806
family
186828
7
node781.members.0.js

genus
2793
node782.members.0.js
39
117563

node783.members.0.js
species
2397
46124

node784.members.0.js
species
137732
357

4
29393
genus

node786.members.0.js
species
4
29394

node787.members.0.js
genus
1
1470540

genus
2747
1

node789.members.0.js
species
1
2748

29
node790.members.0.js
1385
order
4346

family
1
186818

node792.members.0.js
genus
1372
1

4131
539738
family

node794.members.0.js
153
1378
genus
4131

no rank
6
2624949

species
node796.members.0.js
1785995
1

species
node797.members.0.js
4
2840371

1
2040624
node798.members.0.js
species

1379
2440
node799.members.0.js
species

29391
262
node800.members.0.js
species

node801.members.0.js
species
84135
1270

186817
node802.members.0.js
4
15
family

1
400634
genus

1
2636778
no rank

1
2954544
species
node805.members.0.js

genus
node806.members.0.js
1
150247

2
node807.members.0.js
1386
genus
8

1
1792192
species group

species
node809.members.0.js
293387
1

86661
1
node810.members.0.js
species group

3
653685
species group

species subgroup
2
1938374

1
1390
species
node813.members.0.js

node814.members.0.js
species
1
492670

species subgroup
653388
1

node816.members.0.js
species
260554
1

185979
1
no rank

node818.members.0.js
species
2709784
1

genus
1
2837508

species
node820.members.0.js
189381
1

family
170
node821.members.0.js
1
90964

2803850
2
genus

1
1296
species
node823.members.0.js

1
71237
node824.members.0.js
species

167
genus
1279
36
node825.members.0.js

15
species
29388
node826.members.0.js
12

72758
3
subspecies
node827.members.0.js

species group
2815305
1

283734
1
node829.members.0.js
species

species
node830.members.0.js
246432
2

species
node831.members.0.js
39
1282

species
node832.members.0.js
29385
3

1290
14
node833.members.0.js
species

18
1292
node834.members.0.js
species

species
node835.members.0.js
1280
1

species
node836.members.0.js
37
45972

node837.members.0.js
species
29378
1

69
node838.members.0.js
201174
phylum
72017

class
71379
2426
node839.members.0.js
1760

85007
node840.members.0.js
13
10657
order

family
3
85025

1827
2
node842.members.0.js
3
genus

192944
1
no rank

species
node844.members.0.js
1
2663121

family
85029
1

1
37914
genus

499555
1
node847.members.0.js
species

10633
1653
family

53
node849.members.0.js
1716
genus
10633

43770
1
node850.members.0.js
species

1
2624378
no rank

3074378
1
species
node852.members.0.js

1
38302
node853.members.0.js
species

node854.members.0.js
species
38304
2

3
1979527
species
node855.members.0.js

1
2768834
node856.members.0.js
species

node857.members.0.js
species
1522
61592

1
43769
node858.members.0.js
species

node859.members.0.js
species
2
401472

node860.members.0.js
species
1
1719

4
38284
node861.members.0.js
species

679663
1
species
node862.members.0.js

node863.members.0.js
species
3
1717

43768
9037
species
node864.members.0.js

1
node865.members.0.js
1762
family
6

1866885
1
node866.members.0.js
4
genus

117567
1
species
node867.members.0.js

species
node868.members.0.js
1794
1

species
46351
1

1122247
1
strain
node870.members.0.js

genus
1
1763

1
120793
species group

1767
1
species
node873.members.0.js

family
1
85026

1
2053
genus

species
node876.members.0.js
1004901
1

4
85010
order

family
2070
4

1
1813
genus

no rank
1
2618356

species
node881.members.0.js
1896961
1

1
1835
genus
node882.members.0.js

genus
1
1847

no rank
2619320
1

species
node885.members.0.js
445576
1

1
65496
genus

no rank
1
2644606

1
2072503
node888.members.0.js
species

85004
19
order

family
31953
19

node891.members.0.js
2
1678
genus
7

species
158787
1

1150461
1
node893.members.0.js
strain

node894.members.0.js
species
77635
1

33905
1
species
node895.members.0.js

node896.members.0.js
species
1
762210

1
28025
species

302911
1
subspecies

node899.members.0.js
strain
1316911
1

genus
196082
9

species
9
78258

864564
9
node902.members.0.js
strain

genus
3
196081

3
78259
species

3
1150468
strain
node905.members.0.js

order
1
1643682

family
1
85030

genus
88138
1

no rank
1
2643866

species
node910.members.0.js
1
2851567

35479
2037
order

35479
family
2049
node912.members.0.js
331

14
genus
1069494
node913.members.0.js
1

node914.members.0.js
species
11
1661

2733571
1
node915.members.0.js
species

445930
1
species
node916.members.0.js

genus
2740557
243

species
node918.members.0.js
178339
243

1522056
1
genus

1282737
1
species
node920.members.0.js

11172
node921.members.0.js
1654
genus
25997

2755559
5
species
node922.members.0.js

1
2763540
node923.members.0.js
species

19
2057743
species
node924.members.0.js

node925.members.0.js
species
5
52771

2609248
node926.members.0.js
930
8364
no rank

3
2079536
node927.members.0.js
species

species
377
706438

706439
377
node929.members.0.js
strain

4
1851395
node930.members.0.js
species

712116
5998
node931.members.0.js
species

species
node932.members.0.js
2081702
14

2789425
393
species
node933.members.0.js

712122
635
node934.members.0.js
species

species
node935.members.0.js
2789424
9

node936.members.0.js
species
1
2057798

2744574
4
node937.members.0.js
species

species
node938.members.0.js
675090
1

node939.members.0.js
species
3
2057800

species
node940.members.0.js
1960083
1

node941.members.0.js
species
184
1659

species
node942.members.0.js
20
111015

202
1656
species
node943.members.0.js

node944.members.0.js
species
1274
461393

2
2722820
node945.members.0.js
species

3509
species
544580
node946.members.0.js
3006

503
871541
strain
node947.members.0.js

103621
2
node948.members.0.js
species

1655
1059
species
node949.members.0.js

species
node950.members.0.js
6
52774

1852377
164
node951.members.0.js
species

genus
28263
2

28264
2
node953.members.0.js
species

genus
1
2888879

species
node955.members.0.js
1
2692125

genus
2
2050

2051
2
node957.members.0.js
species

genus
1
2912996

2758440
1
node959.members.0.js
species

genus
2692118
1

1
33007
species
node961.members.0.js

2529408
node962.members.0.js
1364
8884
genus

52773
24
node963.members.0.js
species

1660
5704
node964.members.0.js
species

no rank
1788
node965.members.0.js
7
2691889

3
2794089
species
node966.members.0.js

1778
3059028
node967.members.0.js
species

species
node968.members.0.js
4
131111

genus
76833
2

2609299
2
no rank

species
node971.members.0.js
2
2495645

order
85008
1

1
28056
node973.members.0.js
family

1
2495578
order

family
85033
1

2078948
1
genus

node977.members.0.js
species
1
1891644

767
85009
order

family
85015
8

1
2040
genus

species
node981.members.0.js
1
2663859

1839
3
node982.members.0.js
7
genus

species
node983.members.0.js
1
449461

node984.members.0.js
species
1774216
1

species
node985.members.0.js
1
2849501

node986.members.0.js
species
2483798
1

31957
2
node987.members.0.js
759
family

3
1912215
node988.members.0.js
genus

genus
2
1
node989.members.0.js
72763

no rank
1
2635419

2760310
1
node991.members.0.js
species

node992.members.0.js
11
1912216
genus
345

33011
5
node993.members.0.js
species

species
329
node994.members.0.js
318
1747

11
1734925
subspecies
node995.members.0.js

genus
283
3
node996.members.0.js
2801844

node997.members.0.js
species
1750
276

4
1547448
species
node998.members.0.js

genus
124
2
node999.members.0.js
1743

node1000.members.0.js
species
556499
120

node1001.members.0.js
species
2
1744

85011
26
order

26
family
2062
node1003.members.0.js
3

1883
15
node1004.members.0.js
23
genus

1
2839105
species group

node1006.members.0.js
species
1912
1

35621
1
node1007.members.0.js
species

6
2593676
node1008.members.0.js
no rank

order
21998
node1009.members.0.js
77
85006

85016
2
family

genus
2
node1011.members.0.js
1
1707

no rank
1
2620175

node1013.members.0.js
species
1
3039384

85017
2
family

186188
2
genus

2509459
2
species
node1016.members.0.js

1268
24
node1017.members.0.js
21877
family

genus
1
1742989

2627139
1
no rank

1
3023076
node1020.members.0.js
species

32207
957
node1021.members.0.js
21769
genus

396015
2
species
node1022.members.0.js

2047
13024
node1023.members.0.js
13056
species

node1024.members.0.js
strain
762948
32

species
5979
node1025.members.0.js
5687
43675

292
680646
strain
node1026.members.0.js

node1027.members.0.js
species
1773
172042

2689056
2
no rank

2
2810516
species
node1029.members.0.js

2
1663
genus

no rank
2
235627

2987701
1
species
node1032.members.0.js

2991715
1
species
node1033.members.0.js

genus
67
26
node1034.members.0.js
1269

2856555
30
species
node1035.members.0.js

1
2620948
no rank

1
1179670
node1037.members.0.js
species

species
node1038.members.0.js
3
1270

species
node1039.members.0.js
7
1273

57493
2
node1040.members.0.js
14
genus

species
node1041.members.0.js
2
72000

71999
9
node1042.members.0.js
species

1
1049583
node1043.members.0.js
species

family
15
1
node1044.members.0.js
85023

genus
1
1649457

no rank
2649549
1

2985506
1
node1047.members.0.js
species

genus
1
46352

species
node1049.members.0.js
1
3068634

33882
4
node1050.members.0.js
10
genus

69362
1
node1051.members.0.js
species

1572644
1
species
node1052.members.0.js

4
no rank
2609290
node1053.members.0.js
1

1
2603598
node1054.members.0.js
species

species
node1055.members.0.js
2861281
1

1
1906742
species
node1056.members.0.js

33886
1
genus

33887
1
species
node1058.members.0.js

node1059.members.0.js
genus
1
55968

family
145358
1

genus
1
154116

species
node1062.members.0.js
1
2589797

6
85019
family

4
node1064.members.0.js
1696
genus
6

species
node1065.members.0.js
2
33889

family
11
85020

genus
1
1161125

1
521392
species
node1068.members.0.js

36739
1
genus

1630135
1
species
node1070.members.0.js

genus
9
43668

2623841
6
node1072.members.0.js
9
no rank

3
3069708
species
node1073.members.0.js

4
85021
family

4
genus
53457
1
node1075.members.0.js

2649294
3
no rank

3
2761047
species
node1077.members.0.js

145357
1
family

genus
908935
1

no rank
2649892
1

node1081.members.0.js
species
1
2770551

2805590
1
node1082.members.0.js
2
family

125287
1
node1083.members.0.js
genus

class
569
2
node1084.members.0.js
84998

34
1643822
order

34
1643826
family

32
84162
genus

32
84163
species

node1089.members.0.js
strain
32
469378

644652
1
genus

species
1
471189

657308
1
node1092.members.0.js
strain

genus
84111
1

84112
1
species
node1094.members.0.js

84999
node1095.members.0.js
5
533
order

family
527
node1096.members.0.js
9
1643824

133925
175
genus

172
2638792
no rank

node1099.members.0.js
species
172
712411

3
133926
species

633147
3
node1101.members.0.js
strain

2767353
5
node1102.members.0.js
343
genus

species
333
1382

strain
node1104.members.0.js
333
521095

2978947
5
no rank

3022127
5
node1106.members.0.js
species

84107
1
family

genus
1
102106

no rank
1
2637548

node1110.members.0.js
species
1
2844380

phylum
1297
5

5
188787
class

118964
3
order

183710
3
family

node1115.members.0.js
2
1298
genus
3

species
node1116.members.0.js
1
317577

68933
2
order

family
2
188786

genus
node1119.members.0.js
270
2

phylum
568
203691

203692
568
class

order
568
136

family
568
2845253

node1124.members.0.js
224
157
genus
568

node1125.members.0.js
species
69710
27

124
53419
species

subspecies
node1127.members.0.js
124
69713

2638727
node1128.members.0.js
48
96
no rank

node1129.members.0.js
species
2
1643512

species
node1130.members.0.js
120682
17

16
2563662
species
node1131.members.0.js

1659192
2
node1132.members.0.js
species

2766701
11
species
node1133.members.0.js

species
node1134.members.0.js
221027
2

89
species
158
83
node1135.members.0.js

node1136.members.0.js
strain
999426
2

2
999432
node1137.members.0.js
strain

strain
node1138.members.0.js
2
999431

node1139.members.0.js
species
4
58231

2
409322
node1140.members.0.js
species

1783270
node1141.members.0.js
1
39158
clade

clade
39157
node1142.members.0.js
2
68336

976
node1143.members.0.js
150
39155
phylum

117747
1
class

1
200666
order

1
84566
family

28453
1
genus

no rank
2609468
1

species
node1149.members.0.js
2996035
1

117743
3600
class

3600
order
200644
49
node1151.members.0.js

2762318
node1152.members.0.js
21
56
family

genus
12
28250

node1154.members.0.js
species
12
2497989

genus
1
308865

node1156.members.0.js
species
1
1756149

no rank
16
3
node1157.members.0.js
2782232

genus
13
2
node1158.members.0.js
59732

species
node1159.members.0.js
536441
2

no rank
1
2593645

1
2745153
node1161.members.0.js
species

node1162.members.0.js
species
8
2754694

genus
2
node1163.members.0.js
1
1778601

2630820
1
no rank

2704652
1
species
node1165.members.0.js

59735
4
genus

1585976
4
node1167.members.0.js
species

25
node1168.members.0.js
49546
family
3495

node1169.members.0.js
910
1016
genus
3463

26
node1170.members.0.js
2640652
no rank
320

6
2748316
species
node1171.members.0.js

node1172.members.0.js
species
1316593
20

node1173.members.0.js
species
2545799
172

12
1705617
species
node1174.members.0.js

node1175.members.0.js
species
84
1316596

45243
5
node1176.members.0.js
species

327575
156
species
node1177.members.0.js

602
2708117
species
node1178.members.0.js

node1179.members.0.js
species
819
1017

node1180.members.0.js
species
498
1019

153
1018
node1181.members.0.js
species

genus
1209327
1

species
node1183.members.0.js
1803846
1

genus
2715289
1

no rank
2767187
1

2767188
1
species
node1186.members.0.js

genus
1
52959

1908341
1
species
node1188.members.0.js

genus
104267
1

584609
1
node1190.members.0.js
species

237
1
node1191.members.0.js
3
genus

1306519
1
species
node1192.members.0.js

node1193.members.0.js
species
1
2175091

768503
1
class

order
768507
1

family
563798
1

genus
1
280472

species
280473
1

1
758820
strain
node1199.members.0.js

class
35403
node1200.members.0.js
5
200643

order
35398
515
node1201.members.0.js
171549

2
171550
family

239759
2
genus

2364787
1
node1204.members.0.js
species

328814
1
species

strain
node1206.members.0.js
717959
1

185
family
171551
node1207.members.0.js
2

node1208.members.0.js
21
836
genus
183

species
node1209.members.0.js
393921
1

species
node1210.members.0.js
837
53

no rank
95
2645799

species
node1212.members.0.js
712435
95

node1213.members.0.js
species
322095
4

2
867595
node1214.members.0.js
species

species
node1215.members.0.js
28124
3

node1216.members.0.js
species
4
36874

2005525
818
family

genus
813
195950

5
species
28112
2
node1219.members.0.js

203275
2
node1220.members.0.js
strain

1
1307833
node1221.members.0.js
strain

species
node1222.members.0.js
712710
808

node1223.members.0.js
2
375288
genus
5

2685834
1
node1224.members.0.js
species

node1225.members.0.js
species
823
2

family
33836
node1226.members.0.js
387
171552

2974257
81
genus

species
node1228.members.0.js
76123
52

29
28127
species
node1229.members.0.js

3609
node1230.members.0.js
838
genus
30198

species
node1231.members.0.js
44
282402

489
470565
node1232.members.0.js
species

19
28125
node1233.members.0.js
species

28133
1701
node1234.members.0.js
1791
species

strain
node1235.members.0.js
90
702439

2
2801997
node1236.members.0.js
species

node1237.members.0.js
species
20195
28132

2638335
node1238.members.0.js
5
1909
no rank

2
2913620
node1239.members.0.js
species

652716
1875
species

1875
575614
node1241.members.0.js
strain

2691580
2
node1242.members.0.js
species

23
712471
node1243.members.0.js
species

1
652722
node1244.members.0.js
species

species
node1245.members.0.js
2913616
1

52227
8
species

8
908937
strain
node1247.members.0.js

species
node1248.members.0.js
1033
1177574

27
28128
node1249.members.0.js
species

species
node1250.members.0.js
28137
40

589437
118
species

node1252.members.0.js
strain
118
1236518

node1253.members.0.js
species
28129
562

species
37
589436

strain
node1255.members.0.js
37
1236517

28131
298
node1256.members.0.js
315
species

strain
node1257.members.0.js
1122984
17

genus
2884814
2

2133944
2
species
node1259.members.0.js

3168
genus
2974251
10
node1260.members.0.js

species
7
node1261.members.0.js
5
165179

2
537011
node1262.members.0.js
strain

species
node1263.members.0.js
28135
3149

77095
2
species
node1264.members.0.js

2005473
2
family

genus
1
2815786

2606626
1
species
node1267.members.0.js

genus
2518495
1

no rank
node1269.members.0.js
2649562
1

family
2005519
1

397864
1
genus
node1271.members.0.js

2
2005520
family

2
156973
genus

species
node1274.members.0.js
2
45254

2
node1275.members.0.js
815
family
37

genus
node1276.members.0.js
909656
1

genus
34
3
node1277.members.0.js
816

no rank
139043
1

node1279.members.0.js
species
162156
1

28113
11
species
node1280.members.0.js

4
817
species
node1281.members.0.js

1
820
species
node1282.members.0.js

1
2646097
no rank

1
2763022
species
node1284.members.0.js

2
674529
species
node1285.members.0.js

node1286.members.0.js
species
28119
1

9
2650157
node1287.members.0.js
species

species
node1288.members.0.js
1
246787

2323
332
no rank

332
1783234
clade

332
phylum
95818
78
node1291.members.0.js

class
225
2093818

2093819
225
order

family
225
2093822

15
node1295.members.0.js
2093823
genus
225

species
node1296.members.0.js
28
2093824

species
node1297.members.0.js
2572088
29

no rank
2725944
153

species
node1299.members.0.js
153
2899133

29
genus
2905967
8
node1300.members.0.js

node1301.members.0.js
species
2841264
10

no rank
11
2986071

3118470
11
species
node1303.members.0.js

superkingdom
1
2157

1
28890
phylum

clade
2290931
1

class
1
183963

order
2235
1

1
1963268
family

203135
1
genus

2878391
1
node1311.members.0.js
species
