# Supplementary material for: Metagenomic Analysis of the Buccal Microbiome by Nanopore Sequencing Reveals Structural Differences in the Microbiome of a Patient with Molar Incisor Hypomineralization (MIH) Compared to a Healthy Child—Case Study
Source: Int J Mol Sci. 2024 Dec 6;25(23):13143. doi: 10.3390/ijms252313143 (PMC11642311; doi:10.3390/ijms252313143)
Supplement: Supplementary file 1 [file ijms-25-13143-s001.zip › Figure S2. Epithelium_MIH_krona_plot.html]

Javascript must be enabled to view this page.

members
magnitude
magnitudeUnassigned
count
unassigned
taxon
rank

Epithelium\_MIH\_non\_human\_reads\_kraken\_out

1534449
node0.members.0.js
1995

405197
node1.members.0.js

7893
superkingdom
node2.members.0.js
311163
2759

2698737
6
clade

33630
6
clade

6
phylum
5794

5
class
1280412

5
subclass
5796

75739
5
order

423054
5
suborder

family
5
5809

genus
5
5810

5
species
5811

strain
node13.members.0.js
5
508771

1
class
422676

order
1
5863

1
family
32594

5864
genus
1

node18.members.0.js
1
species
5866

2611352
clade
29

29
phylum
33682

5653
29
class

29
subclass
2704647

order
29
2704949

family
29
5654

subfamily
29
1286322

29
genus
5658

38568
29
subgenus

species group
29
38582

29
species
5665

929439
strain
node30.members.0.js
29

33154
node31.members.0.js
303235
14
clade

4751
kingdom
4

4
subkingdom
451864

phylum
4
4890

clade
4
716545

147538
subphylum
2

716546
clade
2

clade
2
715989

147550
2
class

1
subclass
222544

639021
1
order

2528436
1
family

48558
1
genus

1
species
318829

242507
strain
node45.members.0.js
1

222543
1
subclass

1028384
order
1

family
1
681950

5455
1
genus

no rank
1
2707350

34406
1
node51.members.0.js
species

147537
2
subphylum

class
2
4891

order
2
4892

2
clade
2916678

family
2
766764

2
clade
1535325

5475
genus
2

5476
species
2

237561
strain
node60.members.0.js
2

33208
kingdom
303217

6072
clade
303217

clade
303217
33213

33511
clade
303217

7711
303217
phylum

89593
subphylum
303217

clade
303217
7742

303217
clade
7776

117570
clade
303217

117571
clade
303217

8287
303217
superclass

1338369
303217
clade

303217
clade
32523

32524
clade
303217

40674
303217
class

303217
clade
32525

clade
303217
9347

1437010
clade
303217

303217
superorder
314146

9443
order
303217

303217
suborder
376913

314293
infraorder
303217

9526
303217
parvorder

superfamily
303217
314295

9604
303217
family

207598
subfamily
303217

genus
303217
9605

species
node88.members.0.js
303217
9606

815082
node89.members.0.js
4802
superkingdom
2

node90.members.0.js
507389
561
clade
1783272

1239
369544
node91.members.0.js
phylum
630

1
no rank
33974

genus
1
1930845

1871025
species
1
node94.members.0.js

91061
node95.members.0.js
360929
1742
class

node96.members.0.js
36274
243
order
1385

2
family
186823

1502725
1
genus

2743772
node99.members.0.js
1
species

1129704
genus
1

species
node101.members.0.js
1
2055160

6
family
186818

genus
2
1372

node104.members.0.js
1
species
2213202

2662419
no rank
1

2058136
node106.members.0.js
1
species

160795
1
node107.members.0.js
genus

1569
1
genus

2647733
1
no rank

1
node110.members.0.js
species
1930546

genus
1
648800

2637870
1
no rank

species
1
node113.members.0.js
2048654

1
genus
1649

species
node115.members.0.js
1
33946

90964
2
family
node116.members.0.js
35

genus
11
node117.members.0.js
32
1279

586733
species
1
node118.members.0.js

1292
species
3
node119.members.0.js

1282
1
node120.members.0.js
species

species
1
node121.members.0.js
1288

1280
6
node122.members.0.js
species

45972
species
2
node123.members.0.js

species group
1
2815305

283734
1
node125.members.0.js
species

1293
species
node126.members.0.js
1

29379
node127.members.0.js
1
species

1290
species
3
node128.members.0.js

node129.members.0.js
1
species
61015

2803850
1
genus

1
no rank
2803851

3034233
species
node132.members.0.js
1

539738
35961
family

1378
35961
node134.members.0.js
1357
genus

1379
33576
node135.members.0.js
species

29391
species
node136.members.0.js
482

node137.members.0.js
417
species
84135

2624949
node138.members.0.js
129
7
no rank

node139.members.0.js
2
species
1785995

2040624
1
node140.members.0.js
species

species
119
node141.members.0.js
2840371

186817
24
node142.members.0.js
family
10

1
genus
node143.members.0.js
2
2800373

node144.members.0.js
1
species
412384

1
node145.members.0.js
genus
129337

genus
2
2675232

220684
1
node147.members.0.js
species

no rank
1
2675272

3070680
node149.members.0.js
1
species

1386
genus
1
node150.members.0.js
9

4
species group
653685

node152.members.0.js
2
species
1423

1938374
1
species subgroup

1390
node154.members.0.js
1
species

species subgroup
1
653388

260554
species
1
node156.members.0.js

86661
1
species group
node157.members.0.js
4

species
1
1396

1003239
strain
node159.members.0.js
1

1
species
2
node160.members.0.js
1428

1444
1
node161.members.0.js
no rank

1
family
186824

1677050
1
genus

1
node164.members.0.js
species
1471761

family
2
186822

2
genus
44249

1406
species
1
node167.members.0.js

species
1
node168.members.0.js
162209

1474
order
322913
node169.members.0.js
186826

186827
3
family
node170.members.0.js
5797

genus
1
1375

51665
species
1
node172.members.0.js

46123
5792
genus

2857
species
5792
node174.members.0.js
46125

node175.members.0.js
2935
strain
592010

genus
1
2862144

node177.members.0.js
1
species
87458

node178.members.0.js
302295
family
245
1300

node179.members.0.js
7
2
genus
1357

node180.members.0.js
4
2
species
1358

node181.members.0.js
2
subspecies
1360

1359
1
node182.members.0.js
species

1301
genus
177309
302043
node183.members.0.js

1335
node184.members.0.js
3
species

4
node185.members.0.js
species
29389

species
node186.members.0.js
3
1329

1501662
species
node187.members.0.js
8

node188.members.0.js
1
species
1111760

2490633
species
263
node189.members.0.js

16587
node190.members.0.js
species
16295
1313

17
node191.members.0.js
strain
189423

5
node192.members.0.js
strain
869304

1
node193.members.0.js
strain
512566

869216
strain
node194.members.0.js
5

1159083
17
node195.members.0.js
strain

strain
14
node196.members.0.js
488221

525381
1
node197.members.0.js
strain

697283
strain
node198.members.0.js
2

488222
node199.members.0.js
19
strain

strain
node200.members.0.js
161
487214

strain
node201.members.0.js
1
869311

1130804
39
node202.members.0.js
strain

487213
2
node203.members.0.js
strain

516950
8
node204.members.0.js
strain

41
node205.members.0.js
species
33040

1309
408
species
node206.members.0.js
412

strain
node207.members.0.js
1
1225197

3
node208.members.0.js
strain
511691

361101
node209.members.0.js
4
species

node210.members.0.js
76
species
2781599

species
node211.members.0.js
2
1888195

1814128
species
1
node212.members.0.js

node213.members.0.js
2
species
2173853

400065
node214.members.0.js
2
species

2382163
species
node215.members.0.js
25

80
node216.members.0.js
77
species
1307

945704
node217.members.0.js
2
strain

1214179
1
node218.members.0.js
strain

1318
node219.members.0.js
414
338
species

1114965
strain
46
node220.members.0.js

760570
strain
30
node221.members.0.js

species
12
node222.members.0.js
1234680

node223.members.0.js
1
species
254785

2686210
species
node224.members.0.js
1

1917441
species
node225.members.0.js
8

45634
species
284
344
node226.members.0.js

889201
strain
node227.members.0.js
59

strain
node228.members.0.js
1
1302863

1825069
3
node229.members.0.js
species

1303
species
8459
node230.members.0.js
9525

1077464
subspecies
node231.members.0.js
179

292
node232.members.0.js
strain
927666

1458253
node233.members.0.js
145
subspecies

node234.members.0.js
450
subspecies
1891914

684066
species
node235.members.0.js
125

671232
48
species group
588
node236.members.0.js

266
node237.members.0.js
255
species
1338

862967
strain
node238.members.0.js
5

node239.members.0.js
6
strain
862966

76860
species
11
node240.members.0.js

species
node241.members.0.js
263
1328

species
2
node242.members.0.js
197614

6
node243.members.0.js
species
82348

71
species
74
node244.members.0.js
1308

strain
node245.members.0.js
1
767463

1051074
node246.members.0.js
2
strain

68892
species
node247.members.0.js
382

433
node248.members.0.js
species
113107

no rank
3216
12287
node249.members.0.js
2608887

species
318
node250.members.0.js
2759692

3098075
species
node251.members.0.js
37

2610896
node252.members.0.js
65
species

2975324
32
node253.members.0.js
species

2975349
node254.members.0.js
345
species

3098076
species
node255.members.0.js
122

species
27
node256.members.0.js
1759399

2972784
747
node257.members.0.js
species

2954545
species
2
node258.members.0.js

1902136
node259.members.0.js
394
species

2763068
63
node260.members.0.js
species

712623
species
435
node261.members.0.js

1839799
species
node262.members.0.js
49

species
node263.members.0.js
1936
2598453

2954513
species
node264.members.0.js
7

node265.members.0.js
4
species
2598457

species
node266.members.0.js
256
3077723

node267.members.0.js
148
species
3038077

215
node268.members.0.js
species
712624

751
node269.members.0.js
species
712633

3077584
node270.members.0.js
2051
species

2576376
species
1067
node271.members.0.js

366
species
790
node272.members.0.js
1304

strain
3
node273.members.0.js
347253

strain
node274.members.0.js
39
1048332

1074494
strain
node275.members.0.js
1

381
node276.members.0.js
strain
1200793

node277.members.0.js
58
species
1156431

species
10
11
node278.members.0.js
315405

53354
subspecies
1

node280.members.0.js
1
strain
990317

119603
species group
1
node281.members.0.js
21

1
species
13
node282.members.0.js
1336

40041
node283.members.0.js
12
subspecies
7

1051072
node284.members.0.js
5
strain

7
node285.members.0.js
2
species
1334

node286.members.0.js
5
subspecies
4
119602

strain
1
node287.members.0.js
759913

4
node288.members.0.js
species
1310

node289.members.0.js
3
species
1345

1346
species
2
node290.members.0.js

node291.members.0.js
7928
7633
species
1305

388919
node292.members.0.js
295
strain

1
node293.members.0.js
species
1811193

1433513
species
6164
node294.members.0.js

1340
species
node295.members.0.js
2

257758
2851
species

2851
node297.members.0.js
strain
1054460

species
1
150055

strain
1
node299.members.0.js
1076934

2819619
node300.members.0.js
2912
species

node301.members.0.js
57814
53547
species
28037

strain
4267
node302.members.0.js
365659

1314
20
node303.members.0.js
19
species

1
serotype
301451

1
node305.members.0.js
strain
186103

1302
species
node306.members.0.js
942

species
91
node307.members.0.js
1343

species
node308.members.0.js
6
102886

1311
22
species
24
node309.members.0.js

2
node310.members.0.js
strain
1309807

node311.members.0.js
3369
species
2993430

59310
1
node312.members.0.js
species

node313.members.0.js
58
4
family
33958

2767889
1
genus

1
node315.members.0.js
species
1607

genus
19
2742598

node317.members.0.js
1
species
1598

1613
species
18
node318.members.0.js

1243
genus
1

1
species
1245

33967
subspecies
node321.members.0.js
1

46255
1
genus

1583
species
1
node323.members.0.js

2767890
genus
1

1296540
1
node325.members.0.js
species

2759736
7
genus

47715
species
node327.members.0.js
2

1597
5
node328.members.0.js
species

genus
1
2767887

species
node330.members.0.js
1
1624

genus
1
2767893

1581
1
node332.members.0.js
species

1
genus
2767842

1
node334.members.0.js
species
1590

genus
20
1578

node336.members.0.js
1
species
2976676

1584
14
node337.members.0.js
12
species

249265
2
node338.members.0.js
subspecies

2
node339.members.0.js
species
2269374

147802
species
3
node340.members.0.js

2767879
genus
1

species
node342.members.0.js
1
1074467

81852
family
4
node343.members.0.js
23

1350
node344.members.0.js
15
genus
6

3
node345.members.0.js
species
1352

1353
species
node346.members.0.js
1

species
3
node347.members.0.js
1351

2
node348.members.0.js
species
44008

node349.members.0.js
4
genus
2737

74
family
node350.members.0.js
13266
186828

117563
13178
node351.members.0.js
169
genus

node352.members.0.js
2129
species
46124

137732
10880
node353.members.0.js
species

191769
genus
1

2632303
no rank
1

1
node356.members.0.js
species
1911586

29393
genus
9

species
9
node358.members.0.js
29394

1470540
node359.members.0.js
3
1
genus

species
node360.members.0.js
2
1868794

1
genus
2747

species
1
node362.members.0.js
2751

node363.members.0.js
7817
4
class
909932

909929
order
64

family
node365.members.0.js
1
1843490

1843491
63
family

970
17
genus
63
node367.members.0.js

2754044
species
node368.members.0.js
2

node369.members.0.js
30
no rank
12
2637378

1884263
species
node370.members.0.js
7

712528
5
node371.members.0.js
species

712538
node372.members.0.js
3
species

713030
3
node373.members.0.js
species

135079
node374.members.0.js
3
species

69823
11
node375.members.0.js
species

7749
order
1843489

family
7749
31977

39948
2
genus

39950
species
2
node379.members.0.js

29465
genus
943
node380.members.0.js
7747

29466
5464
species
node381.members.0.js
5724

1316254
node382.members.0.js
260
strain

species
7
node383.members.0.js
248315

species
node384.members.0.js
217
39777

203
node385.members.0.js
species
423477

2630086
no rank
282

281
node387.members.0.js
species
2682455

1096935
species
node388.members.0.js
1

39778
113
node389.members.0.js
species

node390.members.0.js
258
species
2682456

node391.members.0.js
73
class
3
186801

3082720
2
order
23
node392.members.0.js

186804
family
3

1870884
3
genus

1496
3
node395.members.0.js
species

family
6
3118655

genus
6
44259

6
species
143361

node399.members.0.js
6
strain
546269

3030910
family
12

12
genus
86331

species
1
node402.members.0.js
114528

species
9
node403.members.0.js
114527

species
2
node404.members.0.js
86332

186802
order
5

31979
node406.members.0.js
3
1
family

genus
2
1485

species
node408.members.0.js
1
1491

238834
node409.members.0.js
1
species

1
family
216572

genus
1
3079925

3029272
1
node412.members.0.js
species

538999
1
no rank

539000
1
family

1
genus
73918

1
node416.members.0.js
species
554949

42
order
3085636

family
2
42
node418.members.0.js
186803

1
genus
1843210

species
node420.members.0.js
1
2696063

1164882
genus
8
node421.members.0.js
39

617123
species
1
node422.members.0.js

2490855
node423.members.0.js
30
species

526524
9
class

526525
node425.members.0.js
9
1
order

8
family
128827

123375
genus
8

102148
species
node428.members.0.js
8

1737404
class
1
85
node429.members.0.js

order
84
1737405

1570339
84
node431.members.0.js
1
family

31983
14
genus

72026
14
node433.members.0.js
species

3
genus
162289

node435.members.0.js
3
species
54005

65
genus
543311

33033
65
node437.members.0.js
species

1
genus
150022

1260
1
node439.members.0.js
species

137146
node440.members.0.js
95
phylum
201174

class
59
84998

84999
58
order

1643824
58
node443.members.0.js
family
1

2767327
1
genus

species
node445.members.0.js
1
82135

2767353
genus
1
28
node446.members.0.js

26
species
1382

521095
26
node448.members.0.js
strain

1
no rank
2978947

3022127
species
1
node450.members.0.js

28
genus
133925

26
no rank
2638792

712411
species
26
node453.members.0.js

133926
2
species

633147
strain
2
node455.members.0.js

order
1
1643822

family
1
1643826

84162
1
genus

84163
species
1

469378
strain
1
node460.members.0.js

136992
node461.members.0.js
3829
class
1760

11
order
85004

31953
11
family

genus
7
196082

7
species
78258

strain
node466.members.0.js
7
864564

196081
1
genus

1
species
78259

strain
node469.members.0.js
1
1150468

1678
1
genus
3
node470.members.0.js

1686
1
species

1447716
node472.members.0.js
1
strain

species
1
216816

1
node474.members.0.js
subspecies
1679

85008
order
6

28056
2
family
node476.members.0.js
6

genus
2
node477.members.0.js
3
1873

1
no rank
2617518

3061627
species
node479.members.0.js
1

genus
1
node480.members.0.js
1865

85007
order
15
node481.members.0.js
2721

2805586
1
family

1847725
genus
1

species
node484.members.0.js
1
1528099

1
family
85029

37914
1
genus

2617939
1
no rank

node488.members.0.js
1
species
712270

family
2
85026

2053
2
genus

1004901
species
node491.members.0.js
2

85025
6
family

1
genus
6
node493.members.0.js
1827

node494.members.0.js
2
no rank
1
192944

1
node495.members.0.js
species
2663121

species
node496.members.0.js
3
103816

1762
family
6

3
node498.members.0.js
1
genus
1763

node499.members.0.js
1
species
1719132

77643
species group
1

species
node501.members.0.js
1
1773

1866885
3
genus

1766
1
node503.members.0.js
species

1
node504.members.0.js
species
134601

1
node505.members.0.js
species
1792

family
2689
1653

node507.members.0.js
2689
genus
20
1716

2624378
no rank
1

node509.members.0.js
1
species
3062461

38304
species
1
node510.members.0.js

1979527
node511.members.0.js
1
species

3
node512.members.0.js
species
1717

2080740
species
node513.members.0.js
3

species
1
38305

1224164
1
node515.members.0.js
strain

43769
species
1
node516.members.0.js

43768
species
211
node517.members.0.js

node518.members.0.js
2447
species
61592

1
family
85028

genus
1
2060

no rank
1
2633480

2929795
species
1
node522.members.0.js

1
order
622452

family
1
83778

33981
1
genus

species
1
131568

strain
1
node527.members.0.js
266940

node528.members.0.js
5954
3
order
85009

4
family
85015

2
genus
3
node530.members.0.js
1839

no rank
1
2615069

1
node532.members.0.js
species
2895565

genus
1
2040

species
node534.members.0.js
1
2663859

31957
node535.members.0.js
5947
family
14

1278221
genus
1

species
node537.members.0.js
1
675864

72763
node538.members.0.js
3
1
genus

2635419
no rank
1

2760310
1
node540.members.0.js
species

399497
species
1
node541.members.0.js

genus
1
2919589

1871034
1
node543.members.0.js
species

33
genus
1743

1744
node545.members.0.js
2
species

119981
species
6
node546.members.0.js

556499
25
node547.members.0.js
species

genus
59
5872
node548.members.0.js
2801844

species
56
node549.members.0.js
1547448

node550.members.0.js
5757
species
1750

1912216
23
genus

node552.members.0.js
7
species
33010

1747
16
node553.members.0.js
species
15

1734925
1
node554.members.0.js
subspecies

85012
2
node555.members.0.js
order
1

family
1
83676

2013
1
genus

280236
species
1

1235441
node559.members.0.js
1
strain

85010
order
3

2070
family
3

genus
1
1813

1804986
node563.members.0.js
1
species

2
genus
1847

2619320
no rank
1

species
node566.members.0.js
1
1641402

37331
node567.members.0.js
1
species

order
23
85011

2062
node569.members.0.js
23
family
2

genus
16
node570.members.0.js
21
1883

no rank
1
node571.members.0.js
2593676

species
node572.members.0.js
3
59299

species
1
node573.members.0.js
116188

85006
92400
node574.members.0.js
146
order

85021
2
family

53457
2
genus

1
node577.members.0.js
species
857417

1
node578.members.0.js
species
262209

1
node579.members.0.js
family
2805590

92209
node580.members.0.js
family
65
1268

32207
92129
node581.members.0.js
genus
4526

43675
node582.members.0.js
2962
species
2811

680646
strain
151
node583.members.0.js

node584.members.0.js
46917
46827
species
2047

762948
90
node585.members.0.js
strain

species
node586.members.0.js
1
169480

37923
1
node587.members.0.js
species

species
node588.members.0.js
37722
172042

1
genus
1742991

species
node590.members.0.js
1
98671

node591.members.0.js
1
genus
1742992

57493
genus
1
node592.members.0.js
5

species
node593.members.0.js
1
71999

72000
species
3
node594.members.0.js

1269
genus
5
node595.members.0.js
6

species
node596.members.0.js
1
574650

2
genus
1742993

2
no rank
2647000

species
node599.members.0.js
2
2851598

1331736
2
family

626119
1
genus

1
node602.members.0.js
species
2897773

genus
1
2828348

species
1
node604.members.0.js
2675754

3
node605.members.0.js
1
family
85017

1
genus
2
node606.members.0.js
186188

2509459
node607.members.0.js
1
species

1
family
85022

43673
genus
1

43674
species
1
node610.members.0.js

family
12
node611.members.0.js
21
85023

genus
1
node612.members.0.js
5
33882

2609290
node613.members.0.js
4
no rank
1

2763257
species
node614.members.0.js
1

1
node615.members.0.js
species
2861280

species
node616.members.0.js
1
3049073

1
genus
33877

node618.members.0.js
1
species
1389020

genus
1
node619.members.0.js
2
46352

3068634
species
1
node620.members.0.js

110932
1
genus

species
1
node622.members.0.js
150026

family
3
145357

3
genus
57495

1274
species
node625.members.0.js
3

3
family
85020

1
genus
36739

1667168
node628.members.0.js
1
species

2
node629.members.0.js
genus
1
43668

2623841
1
no rank

3023517
species
1
node631.members.0.js

85016
node632.members.0.js
6
1
family

2
genus
665568

node634.members.0.js
2
species
1138587

1707
3
node635.members.0.js
1
genus

2
node636.members.0.js
species
2968085

85019
family
1

genus
node638.members.0.js
1
1696

145358
2
family

154116
genus
2

no rank
2
2626815

species
1
node642.members.0.js
2917725

node643.members.0.js
1
species
3120520

32040
order
2037

2049
222
family
node645.members.0.js
32040

10
genus
28263

999183
species
node647.members.0.js
10

2050
genus
11

11
node649.members.0.js
species
2051

genus
6
2740557

178339
species
node651.members.0.js
6

genus
2
9
node652.members.0.js
1069494

3
node653.members.0.js
species
2733571

1661
node654.members.0.js
2
species

445930
2
node655.members.0.js
species

genus
1
1653174

1
node657.members.0.js
species
1870995

genus
1
1522056

node659.members.0.js
1
species
1282737

1654
19113
genus
node660.members.0.js
31324

species
3
node661.members.0.js
2057800

6
node662.members.0.js
species
2321394

node663.members.0.js
5
species
52771

2560010
node664.members.0.js
6
species

675090
2
node665.members.0.js
species

node666.members.0.js
1
species
1960083

2722820
species
node667.members.0.js
3

461393
species
node668.members.0.js
1368

2755559
species
node669.members.0.js
26

1656
node670.members.0.js
1759
species

1852377
node671.members.0.js
4
species

1659
44
node672.members.0.js
species

2737173
species
16
node673.members.0.js

2609248
node674.members.0.js
2279
410
no rank

1851395
species
13
node675.members.0.js

species
node676.members.0.js
412
712116

2789424
species
node677.members.0.js
1

species
node678.members.0.js
79
2789425

706438
species
1278

strain
node680.members.0.js
1278
706439

6
node681.members.0.js
species
2079536

node682.members.0.js
80
species
712122

1655
species
node683.members.0.js
5602

2744574
species
16
node684.members.0.js

species
878
node685.members.0.js
1043
544580

165
node686.members.0.js
strain
871541

52774
16
node687.members.0.js
species

2057743
species
3
node688.members.0.js

node689.members.0.js
6
species
103621

species
node690.members.0.js
3
111015

genus
20
2692118

33007
20
node692.members.0.js
species

node693.members.0.js
435
genus
51
2529408

species
node694.members.0.js
1
131111

2691889
no rank
50

3059028
48
node696.members.0.js
species

node697.members.0.js
2
species
2794089

52773
node698.members.0.js
4
species

species
329
node699.members.0.js
1660

genus
1
2692113

595468
1
node701.members.0.js
species

2495578
order
1

85033
family
1

2078948
1
genus

species
1
node705.members.0.js
1891644

1643682
1
order

1
node707.members.0.js
family
85030

544448
137
node708.members.0.js
1
phylum

2
class
31969

order
1
186328

2131
1
family

1
genus
2132

2139
node713.members.0.js
1
species

order
1
186329

family
1
2146

33926
genus
1

species group
1
85627

35780
1
node718.members.0.js
species

order
134
2790996

2895623
21
family
134
node720.members.0.js

genus
4
2767358

node722.members.0.js
3
species
2112

1
node723.members.0.js
species
28903

108
genus
2923352

29562
node725.members.0.js
108
species

2895509
genus
1

2124
node727.members.0.js
1
species

phylum
1
1297

188787
class
1

order
1
118964

183710
1
family

1
genus
1298

32062
node733.members.0.js
1
species

phylum
8476
32066

203490
class
8476

8476
node736.members.0.js
15
order
203491

1129771
node737.members.0.js
5818
family
16

32067
5732
node738.members.0.js
1999
genus

157687
node739.members.0.js
114
species

369
node740.members.0.js
no rank
24
2633022

species
node741.members.0.js
11
712361

species
35
node742.members.0.js
712362

node743.members.0.js
137
species
712357

species
node744.members.0.js
14
1785996

712368
species
node745.members.0.js
19

129
node746.members.0.js
species
3058373

157688
223
node747.members.0.js
species

536
node748.members.0.js
species
109328

40542
species
47

strain
47
node750.members.0.js
523794

554406
species
2422
node751.members.0.js

157691
22
node752.members.0.js
species

2755140
genus
70

157692
70
node754.members.0.js
species

203492
2643
family

node756.members.0.js
2643
990
genus
848

3
species
node757.members.0.js
6
1583098

1307442
2
node758.members.0.js
strain

1307443
strain
node759.members.0.js
1

2
node760.members.0.js
1
species
859

143387
1
node761.members.0.js
subspecies

species
1423
node762.members.0.js
76857

node763.members.0.js
1
species
285729

76859
species
node764.members.0.js
49

species
2
node765.members.0.js
860

9
species
11
node766.members.0.js
155615

1307427
2
node767.members.0.js
strain

2663009
40
node768.members.0.js
species

1
species
849

469615
strain
1
node770.members.0.js

2648384
no rank
1

671211
species
node772.members.0.js
1

117
node773.members.0.js
14
species
851

node774.members.0.js
103
1
subspecies
76856

node775.members.0.js
102
strain
525283

1224
phylum
2109
node776.members.0.js
279042

1356
class
node777.members.0.js
218749
1236

135615
1101
order

family
1
1101
node779.members.0.js
868

2717
1100
node780.members.0.js
genus
25

94
no rank
2648856

2866573
species
94
node782.members.0.js

species
node783.members.0.js
981
2718

1240482
1
order

1240483
family
1

1193503
1
genus

1196095
species
node787.members.0.js
1

216058
order
135625

712
216058
node789.members.0.js
family
5340

109471
genus
1

1
node791.members.0.js
species
109472

1960084
genus
18

species
node793.members.0.js
7
2778911

1796644
species
node794.members.0.js
11

1249016
genus
1

1032623
species
1
node796.members.0.js

724
208171
node797.members.0.js
39813
genus

2609962
no rank
3902

node799.members.0.js
3902
species
712310

735
node800.members.0.js
2944
species

727
node801.members.0.js
12440
species
9373

strain
3
node802.members.0.js
281310

strain
16
node803.members.0.js
262728

strain
node804.members.0.js
57
1232659

262727
5
node805.members.0.js
strain

biotype
2974
node806.members.0.js
725

10
node807.members.0.js
strain
1295140

strain
node808.members.0.js
2
862964

node809.members.0.js
1
species
730

197575
26
node810.members.0.js
species

249188
node811.members.0.js
257
species

107387
node812.members.0.js
106401
species
729

862965
986
node813.members.0.js
strain

726
41401
node814.members.0.js
species

155493
3
genus
7
node815.members.0.js

750
species
2
node816.members.0.js

505341
species
2
node817.members.0.js

55
genus
2094023

species
node819.members.0.js
44
738

no rank
11
2629322

2030797
11
node821.members.0.js
species

2
genus
2899790

221402
2
node823.members.0.js
species

9
genus
node824.members.0.js
158
745

753
7
node825.members.0.js
species

747
node826.members.0.js
142
species
128

115545
1
node827.members.0.js
subspecies

44283
subspecies
node828.members.0.js
13

node829.members.0.js
5
2
genus
697331

157673
3
species

node831.members.0.js
3
strain
221988

node832.members.0.js
61
20
genus
713

species
node833.members.0.js
1
254839

species
7
node834.members.0.js
189834

species
node835.members.0.js
1
720

715
7
node836.members.0.js
6
species

754344
no rank
1

754256
node838.members.0.js
1
strain

51049
10
node839.members.0.js
species

716
node840.members.0.js
2
species

13
node841.members.0.js
4
species
718

5
node842.members.0.js
subspecies
202947

202948
subspecies
4
node843.members.0.js

214906
genus
6

731
5
species
6
node845.members.0.js

228400
strain
node846.members.0.js
1

292486
genus
6

728
node848.members.0.js
6
species

75984
genus
21
34
node849.members.0.js

75985
6
node850.members.0.js
species

4
node851.members.0.js
species
85402

85404
2
species

1433287
2
node853.members.0.js
strain

111844
1
node854.members.0.js
species

416916
genus
439
2193
node855.members.0.js

739
node856.members.0.js
77
species

species
950
1058
node857.members.0.js
732

634176
56
node858.members.0.js
strain

985008
node859.members.0.js
52
strain

48
species
node860.members.0.js
49
714

strain
1
node861.members.0.js
272556

no rank
36
node862.members.0.js
570
2639383

63
node863.members.0.js
species
2866570

712150
species
382
node864.members.0.js

species
node865.members.0.js
89
2820817

order
2
21
node866.members.0.js
135614

family
19
32033

genus
1
40323

216778
species
node869.members.0.js
1

338
18
node870.members.0.js
3
genus

339
species
node871.members.0.js
15

135619
3
node872.members.0.js
1
order

28256
family
2
node873.members.0.js

135622
3
order

1
family
267891

58050
1
genus

69539
species
node877.members.0.js
1

267890
family
2

genus
1
2
node879.members.0.js
22

1
species
62322

1
node881.members.0.js
strain
693970

order
10
135623

641
family
3
node883.members.0.js
10

node884.members.0.js
7
genus
1
662

4
no rank
2614977

2819096
node886.members.0.js
1
species

2751178
species
node887.members.0.js
2

species
1
node888.members.0.js
2751179

species group
1
717610

670
species
node890.members.0.js
1

species
1
node891.members.0.js
1381081

29
order
2887326

family
29
468

1
genus
15
node894.members.0.js
469

8
species group
909768

470
node896.members.0.js
8
species
7

1400867
1
node897.members.0.js
strain

1
node898.members.0.js
species
2715163

1
no rank
196816

species
1
node900.members.0.js
2919376

28090
species
node901.members.0.js
1

species
node902.members.0.js
1
465797

52133
species
2

1197884
2
node904.members.0.js
strain

475
2
genus
node905.members.0.js
14

90239
species
node906.members.0.js
1

2
no rank
2685852

2953752
species
node908.members.0.js
2

species
9
node909.members.0.js
34062

1706369
order
1

family
1
1706372

3040948
1
genus

2518994
node913.members.0.js
1
species

72274
order
20

2887365
family
1

2742
genus
1

1
species
1305740

strain
node918.members.0.js
1
626887

135621
family
19

genus
12
18
node920.members.0.js
286

136849
species group
node921.members.0.js
1

321846
node922.members.0.js
1
species

2
species group
136841

287
2
species

1408275
2
node925.members.0.js
strain

196821
1
no rank

3003351
species
1
node927.members.0.js

136843
species group
1

76760
species
node929.members.0.js
1

2901164
1
genus

136846
species group
1

578833
1
species subgroup

316
node933.members.0.js
1
species

91347
144
node934.members.0.js
order
55

family
3
1903412

3
genus
635

67780
3
node937.members.0.js
species
2

634503
strain
1
node938.members.0.js

family
5
1903414

626
genus
1

2632833
no rank
1

3130166
species
1
node942.members.0.js

586
genus
3

no rank
3
2633465

species
node945.members.0.js
1
2949878

node946.members.0.js
1
species
2949758

node947.members.0.js
1
species
2936783

637
1
genus

2879119
1
node949.members.0.js
species

1903411
family
17

genus
1
629

1649845
species group
1

632
1
species

1
node954.members.0.js
strain
1035377

3
node955.members.0.js
genus
613

34037
genus
1
node956.members.0.js
13

2703885
node957.members.0.js
11
species

1510570
1
node958.members.0.js
species

543
node959.members.0.js
63
family
31

191675
no rank
1

84563
1
clade

801
1
genus

2871826
node963.members.0.js
1
species

genus
3
544

1344959
species group
3

node966.members.0.js
3
species
546

genus
1
1081630

species
node968.members.0.js
1
1081631

547
3
genus

354276
2
species group

61645
species
node971.members.0.js
1

1812935
1
node972.members.0.js
species

species
1
node973.members.0.js
881260

561
genus
2
16
node974.members.0.js

species
14
node975.members.0.js
562

no rank
6
2890311

570
1
genus
node977.members.0.js
6

548
species
1
node978.members.0.js

species
4
node979.members.0.js
573

590
2
genus

28901
2
species

59201
2
subspecies

node983.members.0.js
1
no rank
2583588

604
no rank
1

strain
node985.members.0.js
1
1225522

1903409
1
family

53335
genus
1

species
1
node988.members.0.js
472693

2
order
135624

84642
family
2

node991.members.0.js
2
genus
1
642

654
node992.members.0.js
1
species

28211
13
class
node993.members.0.js
41

204457
order
1
7
node994.members.0.js

41297
family
2
node995.members.0.js
5

genus
2
13687

species
node997.members.0.js
1
563996

196159
no rank
1

node999.members.0.js
1
species
3014784

165695
1
genus

no rank
1
node1001.members.0.js
2611147

335929
1
family

2800788
no rank
1
node1003.members.0.js

204455
order
4

family
1
node1005.members.0.js
4
31989

3
node1006.members.0.js
genus
1
265

147645
species
node1007.members.0.js
1

59779
species
node1008.members.0.js
1

node1009.members.0.js
14
3
order
356

1
family
255475

1
genus
414371

no rank
1
2615206

2816454
node1013.members.0.js
1
species

119045
3
family

node1015.members.0.js
3
genus
407

82115
family
1
2
node1016.members.0.js

227290
no rank
1

1
node1018.members.0.js
genus
379

41294
4
family

genus
2
node1020.members.0.js
4
374

no rank
1
node1021.members.0.js
2
2631580

species
1
node1022.members.0.js
3140244

1
family
31993

1
genus
133

1
node1025.members.0.js
species
655015

3108222
order
1

family
1
2066434

171436
1
genus

171437
1
species

node1030.members.0.js
1
strain
1110502

204458
2
order

76892
2
family

41275
2
node1033.members.0.js
genus

58143
node1034.members.0.js
class
318
28216

80840
106
order
7815
node1035.members.0.js

224471
no rank
1
node1036.members.0.js
2

1
genus
1597779

1597781
species
1
node1038.members.0.js

80864
node1039.members.0.js
186
6
family

179
genus
219181

179
no rank
2645081

1658672
species
node1042.members.0.js
179

1
genus
2919468

node1044.members.0.js
1
species
2029117

506
node1045.members.0.js
5
2
family

genus
2
106146

2652177
species
node1047.members.0.js
2

1
genus
29574

29575
species
node1049.members.0.js
1

119060
30
family
node1050.members.0.js
7510

genus
1
1822464

species
1
148447

391038
strain
node1053.members.0.js
1

genus
7442
47670

47671
species
node1055.members.0.js
7442

37
genus
32008

no rank
1
2613784

1494466
1
node1058.members.0.js
species

111527
1
species group

28450
node1060.members.0.js
1
species

87882
35
species group

87883
species
35
node1062.members.0.js

75682
node1063.members.0.js
6
family
1

no rank
3
4
node1064.members.0.js
2895353

149698
1
genus

species
1
node1066.members.0.js
2899220

node1067.members.0.js
1
genus
963

32003
order
1

node1069.members.0.js
1
family
206379

order
107
node1070.members.0.js
50009
206351

2897176
family
1

168470
1
genus

168471
species
1

1
node1074.members.0.js
strain
557598

1499392
1
family

57739
genus
1

no rank
1
2684990

2877939
1
node1078.members.0.js
species

49900
node1079.members.0.js
family
3864
481

3
genus
71

species
node1081.members.0.js
3
72

334107
genus
1

species
node1083.members.0.js
1
153493

41
genus
286
node1084.members.0.js
538

species
12
node1085.members.0.js
2528037

539
233
node1086.members.0.js
species

node1087.members.0.js
5928
genus
102
32257

node1088.members.0.js
17
species
265175

502
species
891
node1089.members.0.js

node1090.members.0.js
30
species
1522312

504
species
3
node1091.members.0.js

505
node1092.members.0.js
4885
species

194195
genus
5

194196
species
2
node1094.members.0.js

species
node1095.members.0.js
3
153491

482
16747
genus
39571
node1096.members.0.js

483
species
node1097.members.0.js
9072

487
node1098.members.0.js
873
species
806

135720
17
serogroup

374833
strain
node1100.members.0.js
13

node1101.members.0.js
4
strain
604162

strain
2
node1102.members.0.js
935599

13
node1103.members.0.js
strain
942513

node1104.members.0.js
15
strain
662598

1172206
node1105.members.0.js
3
strain

strain
node1106.members.0.js
16
935588

935591
node1107.members.0.js
1
strain

species
node1108.members.0.js
218
267212

28449
node1109.members.0.js
419
species

no rank
95
8016
node1110.members.0.js
2623750

655307
species
348
node1111.members.0.js

1
node1112.members.0.js
species
1871106

2912675
species
node1113.members.0.js
3

2972775
22
node1114.members.0.js
species

3077590
species
89
node1115.members.0.js

2937985
species
904
node1116.members.0.js

1871109
node1117.members.0.js
27
species

641148
species
6527

641149
strain
6527
node1119.members.0.js

node1120.members.0.js
1
species
2709396

1470200
species
2
node1121.members.0.js

species
158
161
node1122.members.0.js
485

521006
node1123.members.0.js
2
strain

strain
1
node1124.members.0.js
1247414

326522
species
node1125.members.0.js
3

1853276
3
node1126.members.0.js
species

1853278
species
node1127.members.0.js
5

1091
species
496

strain
1091
node1129.members.0.js
997348

493
node1130.members.0.js
1
species

5
node1131.members.0.js
species
194197

node1132.members.0.js
2
species
492

12
node1133.members.0.js
species
607712

392
species
node1134.members.0.js
476
495

subspecies
node1135.members.0.js
84
88719

486
node1136.members.0.js
331
species
301

489653
strain
30
node1137.members.0.js

species
8
node1138.members.0.js
1815583

33053
78
node1139.members.0.js
species

484
species
node1140.members.0.js
114

species
node1141.members.0.js
4
2912188

490
node1142.members.0.js
1548
species

488
node1143.members.0.js
378
species

2995413
node1144.members.0.js
1
species

2666100
species
node1145.members.0.js
2

3
genus
1193515

2946699
1
node1147.members.0.js
species

species
node1148.members.0.js
2
1196083

299568
genus
6

species
5
node1150.members.0.js
252130

1
node1151.members.0.js
species
1282863

genus
3
2944815

2917790
node1153.members.0.js
3
species

node1154.members.0.js
1
genus
59

2898489
3
genus

2809020
species
3
node1156.members.0.js

genus
226
212742

1056807
species
node1158.members.0.js
226

phylum
46
203691

46
class
203692

order
46
136

46
family
2845253

46
node1163.members.0.js
12
genus
157

69710
node1164.members.0.js
3
species

58231
species
node1165.members.0.js
2

409322
18
node1166.members.0.js
species

2638727
no rank
5

2766701
3
node1168.members.0.js
species

2563662
species
node1169.members.0.js
2

species
node1170.members.0.js
2
158

species
4
53419

subspecies
4
node1172.members.0.js
69713

1783270
clade
13262

68336
1
clade
node1174.members.0.js
13262

976
phylum
302
13261
node1175.members.0.js

3
class
768503

768507
order
3

family
1
563798

246875
1
genus

1
no rank
2641541

species
1
node1181.members.0.js
3097546

1853232
1
family

89966
genus
1

node1184.members.0.js
1
species
748197

1
family
1937968

1937972
1
node1186.members.0.js
genus

10727
class
117743

200644
node1188.members.0.js
10727
545
order

49546
93
family
9611
node1189.members.0.js

1
genus
379068

2632773
1
no rank

1170699
node1192.members.0.js
1
species

52959
2
genus

species
1
node1194.members.0.js
1888915

196858
no rank
node1195.members.0.js
1

3
node1196.members.0.js
genus
2
76831

node1197.members.0.js
1
species
256

2715289
1
genus

2767187
1
no rank

1
node1200.members.0.js
species
2767188

237
24
node1201.members.0.js
genus
13

2906077
species
7
node1202.members.0.js

node1203.members.0.js
1
species
1751056

996
species
node1204.members.0.js
1

3003256
node1205.members.0.js
1
species

2893886
species
1
node1206.members.0.js

1739
genus
node1207.members.0.js
9486
1016

species
node1208.members.0.js
250
45243

327575
species
65
node1209.members.0.js

node1210.members.0.js
209
13
no rank
2640652

species
node1211.members.0.js
84
1316596

9
node1212.members.0.js
species
2748316

2545799
node1213.members.0.js
55
species

13
node1214.members.0.js
species
1316593

species
node1215.members.0.js
35
1705617

3
node1216.members.0.js
species
28188

1019
species
node1217.members.0.js
5724

species
184
node1218.members.0.js
1018

1017
species
669
node1219.members.0.js

2708117
species
node1220.members.0.js
643

143222
genus
1
node1221.members.0.js

2762318
568
node1222.members.0.js
276
family

68
genus
28250

species
node1224.members.0.js
1
28251

2497989
species
67
node1225.members.0.js

59735
1
genus
node1226.members.0.js
37

species
14
node1227.members.0.js
1735111

1585976
species
22
node1228.members.0.js

34084
genus
2
node1229.members.0.js
13

103810
3
node1230.members.0.js
species

8
node1231.members.0.js
species
34085

501783
genus
node1232.members.0.js
3

genus
node1233.members.0.js
1
59734

2782232
44
no rank
node1234.members.0.js
164

59732
109
node1235.members.0.js
genus
86

59733
1
node1236.members.0.js
species

2593645
13
node1237.members.0.js
no rank
5

2983267
species
1
node1238.members.0.js

node1239.members.0.js
1
species
2039166

1
node1240.members.0.js
species
1871047

2478663
species
1
node1241.members.0.js

node1242.members.0.js
1
species
2745153

node1243.members.0.js
1
species
2838877

1
node1244.members.0.js
species
2015076

878220
node1245.members.0.js
1
species

421058
species
node1246.members.0.js
1

2754694
species
node1247.members.0.js
1

node1248.members.0.js
1
species
254

1
node1249.members.0.js
species
192389

species
3
node1250.members.0.js
1265445

651561
node1251.members.0.js
1
species

species
node1252.members.0.js
1
253

2782229
3
genus

2487072
3
node1254.members.0.js
species

genus
3
8
node1255.members.0.js
2782231

species
1
node1256.members.0.js
2878534

2038776
2
node1257.members.0.js
species

2487074
species
1
node1258.members.0.js

266748
species
node1259.members.0.js
1

308865
1
genus

238
node1261.members.0.js
1
species

node1262.members.0.js
5
3
genus
1778601

2500547
1
node1263.members.0.js
species

2630820
node1264.members.0.js
1
no rank

family
3
39782

genus
1
node1266.members.0.js
3
34098

1653831
node1267.members.0.js
2
species

200643
node1268.members.0.js
2228
13
class

2215
node1269.members.0.js
order
119
171549

815
6
node1270.members.0.js
family
1

816
node1271.members.0.js
5
genus
1

1
node1272.members.0.js
species
291645

291644
node1273.members.0.js
1
species

species
2
node1274.members.0.js
28116

2760872
family
1

1
genus
2760873

2590900
node1277.members.0.js
1
species

171552
43
family
node1278.members.0.js
1861

genus
2
257
node1279.members.0.js
2974251

species
255
node1280.members.0.js
28135

2974257
17
genus

28127
1
node1282.members.0.js
species

node1283.members.0.js
16
species
76123

node1284.members.0.js
1544
genus
205
838

28133
58
species
node1285.members.0.js
64

702439
node1286.members.0.js
6
strain

28131
29
species
node1287.members.0.js
31

1122984
node1288.members.0.js
2
strain

589436
1
species

1236517
strain
node1290.members.0.js
1

species
node1291.members.0.js
45
1177574

282402
node1292.members.0.js
1
species

species
node1293.members.0.js
213
470565

28132
species
692
node1294.members.0.js

5
node1295.members.0.js
species
28125

77
node1296.members.0.js
no rank
1
2638335

712471
node1297.members.0.js
7
species

652716
69
species

575614
node1299.members.0.js
69
strain

species
1
52227

908937
strain
node1301.members.0.js
1

28129
species
100
node1302.members.0.js

28128
species
4
node1303.members.0.js

589437
7
species

1236518
strain
7
node1305.members.0.js

28137
species
node1306.members.0.js
98

50
family
2005525

50
genus
195950

28112
1
species
node1309.members.0.js
2

node1310.members.0.js
1
strain
1307833

node1311.members.0.js
48
species
712710

family
1
2005520

156973
1
genus

1
node1314.members.0.js
species
45254

171550
family
1
node1315.members.0.js

176
family
171551

836
genus
25
node1317.members.0.js
176

node1318.members.0.js
21
species
837

no rank
123
2645799

species
node1320.members.0.js
123
712435

7
node1321.members.0.js
species
28124

1853228
class
1

1853229
1
order

family
1
563835

1769012
1
genus

2341117
1
node1326.members.0.js
species

phylum
1
200940

1
class
3031451

1
order
3024411

213121
1
family

1
genus
893

1
node1332.members.0.js
species
1986146

14
no rank
2323

1783234
14
clade

95818
node1335.members.0.js
14
phylum
1

13
class
2093818

order
13
2093819

2093822
family
13

genus
13
2093823

2093824
node1340.members.0.js
11
species

2725944
2
no rank

species
2
node1342.members.0.js
2899133

phylum
1
3018035

class
1
1553900

2024973
order
1

2024974
1
family

genus
1
2838409

2838410
no rank
1

species
1
node1349.members.0.js
2976982

2049
phylum
29547

3031852
class
2049

1
order
235899

1
family
224467

191291
node1354.members.0.js
1
genus

213849
node1355.members.0.js
2048
4
order

2044
family
72294

194
2044
node1357.members.0.js
119
genus

species
9
node1358.members.0.js
200

199
node1359.members.0.js
141
species
134

360104
strain
7
node1360.members.0.js

824
1518
node1361.members.0.js
species

204
212
node1362.members.0.js
species

2593542
1
no rank

1
node1364.members.0.js
species
2983836

44
node1365.members.0.js
species
203

10239
superkingdom
1006

2732004
clade
1

1
kingdom
2732005

2732007
1
phylum

1
class
2732523

1
order
2732554

549779
family
node1372.members.0.js
1

2731341
1005
clade

2731360
kingdom
1005

144
phylum
2731618

3
class
node1376.members.0.js
144
2731619

3044455
family
1
node1377.members.0.js
10

1623304
genus
1

species
1
node1379.members.0.js
72638

genus
node1380.members.0.js
1
1623303

no rank
7
3094845

537874
node1382.members.0.js
7
species

2788787
node1383.members.0.js
122
no rank
9

species
node1384.members.0.js
1
683735

47
node1385.members.0.js
species
644007

28
node1386.members.0.js
species
12402

1701814
1
node1387.members.0.js
species

1448274
node1388.members.0.js
3
species

node1389.members.0.js
28
species
1566990

12348
species
node1390.members.0.js
1

node1391.members.0.js
4
species
1701811

family
9
2842328

1
genus
node1393.members.0.js
9
1982583

species
6
1982584

10747
no rank
6
node1395.members.0.js

1982585
2
species

10748
no rank
2
node1397.members.0.js

861
phylum
2731361

861
class
2731363

548681
861
order

family
861
3044472

subfamily
849
10374

5
genus
node1403.members.0.js
849
10375

3050299
species
844

10376
node1405.members.0.js
844
no rank
760

12509
no rank
84
node1406.members.0.js

subfamily
12
10357

genus
12
40272

species
1
3050297

no rank
node1410.members.0.js
1
32604

3050298
species
11

no rank
node1412.members.0.js
11
10372

superkingdom
6
2157

6
phylum
28890

2290931
5
clade

5
class
183963

2235
order
3
node1417.members.0.js
5

1644056
1
family

1911573
genus
1

node1420.members.0.js
1
species
2876193

1963268
node1421.members.0.js
1
family

2283794
clade
1

183925
1
class

order
1
2158

2159
1
family

2172
1
genus

2173
1
species

strain
node1428.members.0.js
1
420247
